# Early activation of the cardiac CX3CL1/CX3CR1 axis delays β-adrenergic-induced heart failure

**DOI:** 10.1038/s41598-021-97493-z

**Published:** 2021-09-09

**Authors:** M. Flamant, N. Mougenot, E. Balse, L. Le Fèvre, F. Atassi, E. L. Gautier, W. Le Goff, M. Keck, S. Nadaud, C. Combadière, A. Boissonnas, C. Pavoine

**Affiliations:** 1 Sorbonne Université, UPMC Univ Paris 06, INSERM, Institute of Cardiometabolism and Nutrition (ICAN), Team 3, UMR_S ICAN 1166 Team 3, 91 bd de l’hôpital, 75013 Paris, France; 2 Sorbonne Université, UMS28, Plateforme d’Expérimentation Cœur, Muscles, Vaisseaux (PECMV), 75013 Paris, France; 3 Sorbonne Université, UPMC Univ Paris 06, INSERM, Institute of Cardiometabolism and Nutrition (ICAN), UMR_S ICAN 1166 Team 5, 75013 Paris, France; 4 Sorbonne Université, UPMC Univ Paris 06, INSERM, Institute of Cardiometabolism and Nutrition (ICAN), UMR_S ICAN 1166 Team 4, 75013 Paris, France; 5 Sorbonne Université, Inserm, CNRS, Centre d’Immunologie et des Maladies Infectieuses CIMI-Paris, 75013 Paris, France; 6 Medical and Infectious Intensive Care Unit, Bichat hospital, APHP, 46 rue Henri Huchard, 75018 Paris, France; 7 Département Médicaments et Technologies pour la Santé (DMTS), Université Paris-Saclay, CEA, INRAE, SIMoS, 91191 Gif-sur-Yvette, France

**Keywords:** Biological techniques, Cell biology, Immunology, Molecular biology, Physiology, Cardiology, Diseases

## Abstract

We recently highlighted a novel potential protective paracrine role of cardiac myeloid CD11b/c cells improving resistance of adult hypertrophied cardiomyocytes to oxidative stress and potentially delaying evolution towards heart failure (HF) in response to early β-adrenergic stimulation. Here we characterized macrophages (Mφ) in hearts early infused with isoproterenol as compared to control and failing hearts and evaluated the role of upregulated CX3CL1 in cardiac remodeling. Flow cytometry, immunohistology and Mφ-depletion experiments evidenced a transient increase in Mφ number in isoproterenol-infused hearts, proportional to early concentric hypertrophy (ECH) remodeling and limiting HF. Combining transcriptomic and secretomic approaches we characterized Mφ-enriched CD45^+^ cells from ECH hearts as CX3CL1- and TNFα-secreting cells. In-vivo experiments, using intramyocardial injection in ECH hearts of either *Cx3cl1* or *Cx3cr1* siRNA, or *Cx3cr1*^−/−^ knockout mice, identified the CX3CL1/CX3CR1 axis as a protective pathway delaying transition to HF. In-vitro results showed that CX3CL1 not only enhanced ECH Mφ proliferation and expansion but also supported adult cardiomyocyte hypertrophy via a synergistic action with TNFα. Our data underscore the *in-vivo* transient protective role of the CX3CL1/CX3CR1 axis in ECH remodeling and suggest the participation of CX3CL1-secreting Mφ and their crosstalk with CX3CR1-expressing cardiomyocytes to delay HF.

## Introduction

Hemodynamic cardiac stimulation initially induces early hypertrophy, a compensatory response to reduce parietal stress and prevent dysfunction. Chronic hypertrophy associated with apoptosis, fibrosis, inflammation responses and angiogenic alteration triggers transition to heart failure (HF), a major health issue^1^. The activation of the sympathetic nervous system plays a determinant role throughout cardiac remodeling^2–4^ but the mechanisms underlying the transition from early hypertrophy to HF are poorly understood. Therefore, a better understanding of cellular mechanisms elicited during early hypertrophy is needed to prevent the progression to HF or favor recovery^5,6^.

In addition to cardiomyocyte hypertrophy, cardiac hypertrophic remodeling is associated with determinant changes of non-myocyte cardiac cell types, including myeloid cells^7^. Different studies showed that cardiac hypertrophy is a complex inflammatory syndrome^5^ where cardiac macrophages (Mφ) are critical effectors. Cardiac Mφ influence tissue homeostasis, repair and regeneration and modulate cardiac hypertrophy and HF^8,9^. Mφ have been extensively implicated in the inflammatory response to myocardial infarction^10^. A growing body of evidence suggests that they also play a critical role in the pathogenesis of chronic non-ischemic heart remodeling^11,12^. The protective role of resident Mφ has been established in the control of electrical conduction and the clearance of dysfunctional mitochondria^13,14^. However, the mechanisms of activation and the functions of cardiac Mφ are not fully understood in the context of hypertrophic remodeling, with conflicting results as to whether they are beneficial or detrimental to the heart^1–3^. Our recent results suggest a novel protective paracrine impact of cardiac myeloid CD11b/c cells leading to early hypertrophy and improved resistance to oxidative stress^15^.

Using flow cytometry, transcriptomic, and secretomic analysis approaches, the aim of the present study was to isolate and characterize Mφ selectively amplified in early hypertrophy as compared to control and HF hearts, in order to better define their protective mechanism of action. We identify the cardiac CX3CL1/CX3CR1 axis as a protective signaling pathway in early hypertrophy and the potential participation of CX3CL1-secreting Mφ, transiently amplified in ECH hearts, to delay HF.

## Methods

The detailed methods are shown in the Supplementary material online.

### Ethics

Care of the animals and surgical procedures were performed according to the Directive 2010/63/EU of the European Parliament, which had been approved by the Ministry of Agriculture, France, (authorization for surgery C-75-665-R). The project was submitted to the French Ethic Committee CEEA (*Comité d’Ethique en Expérimentation Animale*) and obtained the authorization Ce5/2012/050 and APAFIS#1729-2015-083114195840v8. All experiments were performed in accordance with relevant named guidelines and regulations and in compliance with the ARRIVE guidelines.

Isoflurane was used to anesthetize mice during echocardiography analysis (0.2–0.5%), intramyocardial siRNA injections (1%), and Alzet micropump implantation (2–3%). The adequacy of anesthesia was confirmed by the absence of a reflex response to foot squeeze. Mice were euthanized via cervical dislocation and hearts were isolated for further histological and protein analyses or before cardiomyocyte or macrophage isolations.

### Animals

Experiments were conducted on adult male mice (9–20 week-old) of the following strains: C57BL/6J mice purchased from Janvier Labs (Le-Genest-St-Isle, France); *Cx3cr1*^*-/-*^ mice (C57BL/6J genetic background, as previously reported^16^) bred at Pitié-Salpétrière animal facility.

### In-vivo chronic isoproterenol infusion

Mice were implanted with an osmotic micropump (Alzet, Charles River, L’Arbresles, France) containing either isoproterenol (iso: 30 mg/kg/day) or vehicle for either 14 or 28 days to develop either ECH or HF, respectively, as previously reported^15^. Of note there was a slight but possible variability in the kinetics or in the maximum intensity of cardiac remodeling triggered by iso: for this reason, protocols always included a group of control animals (WT or untreated or control injected) in parallel, to allow comparison. In addition, we observed a reduced or delayed cardiac remodeling and a potential absence of HF at day 25–28 iso in experiments involving repeated injections of either liposomes or PBS: our hypothesis was that repeated injections could generate a low-grade inflammatory response capable of limiting or delaying deleterious cardiac remodeling.

### In-vivo intramyocardial ultrasound-guided transthoracic siRNA delivery in mice

On-target plus Scramble or *Cx3cl1* or *Cx3cr1* siRNA (Dharmacon, Cambridge, UK) were injected by ultrasound-guided transthoracic intramyocardial injection (see Figure S9) in mice, as described in Keck et al.^15^, at day 7 after iso-pump implantation. Echocardiographic parameters were measured regularly as stated.

### Measurement of cardiac parameters

Echocardiography was performed on lightly anesthetized animals under isoflurane (0.2–0.5%) with a probe emitting ultrasounds from 9- to 14-MHz frequency (Vivid7 PRO apparatus; GE Medical System Co), as previously reported^15^.

### Cardiomyocyte isolation and culture

Cardiomyocytes were isolated from adult mice using a simplified Langendorff-free method, as previously reported^17^.

Cardiomyocytes were plated onto laminin-coated wells (5 µg/ml, Roche) at a density of 30,000 total cells/ml in a plating medium (M199 medium (Life Technologies, Courtaboeuf, France)/Joklik medium (1/1 vol/vol) added with 10 mM BDM, 1% penicillin–streptomycin (PS), 1% insulin/transferrin/selenium (ITS) and 5% FBS). They were let to adhere for 3 h before treatments with either CX3CL1 or TNFα or both, conditioned medium (Cmed) from Mφ-enriched adherent CD45^+^cells ± pharmacological inhibitors (with RPMI medium as internal control, prepared as described below), or ± CX3CL1 or TNFR_1_ or TNFR_2_ antibodies (Abs), when stated, in a culture medium (idem plating medium but with only 1.5% FBS) and maintained overnight. Each experimental condition was evaluated in triplicate.

### Measurement of cardiomyocyte hypertrophy

Cardiomyocytes were visualized using brightfield at × 20 magnification and cell width, length and area were measured in at least 300 cells per condition per experiment. Results were the mean of at least three different experiments performed on two cell isolations (using at least 4 different Cmed from adherent CD45^+^cells).

### Isolation of cardiac immune cells for Conditioned media (Cmed) preparation and in-vitro proliferation assay

After perfusion with PBS, the mice heart was excised and digested in HBSS medium containing 2.5 mg/ml collagenase D (Roche, Meylan, France) for 30 min at 37 °C, with stirring. Erythrocytes were lysed by using red blood cell lysis buffer (MiltenyiBiotec, Paris, France). Cardiac CD45 cells were isolated by centrifugation, enriched using an anti-CD45 antibody coupled to magnetic beads (MiltenyiBiotec, Paris, France) and all CD45^+^ cells isolated from each heart were systematically seeded in 2 wells from a 48 multiwell plate (final volume 500 µl in a RPMI medium (Life Technologies, Courtaboeuf, France) supplemented with 10 mmol/LHepes). After 3 h of adhesion, the medium was renewed (this allowed elimination of non-adherent cells such as lymphocytes or NKT cells) and adherent cells (containing an average of 64.2 ± 3.5% of CD64 positive cells) were incubated overnight before collection of their conditioned medium (Cmed). The Cmed was concentrated 3 times on Amicon 3 kDa Ultra centrifugal filter (Millipore), kept at − 80 °C until in-vitro studies on cardiomyocyte hypertrophy and used at a 1/20 final dilution. RPMI medium treated in parallel and in the same way than Cmed was used as internal control. Cytokines and chemokines in the Cmed were quantified using a bio-plex immunoassay (Biorad, Marnes-La-Coquette, France). For in-vitro proliferation assays, Mφ were kept in culture for 48 h in the presence or absence of CX3CL1 (50 ng/ml) before fixation in paraformaldehyde and fluorescent staining.

### Quantification of macrophage proliferation

For BrdU incorporation assays, mice were injected intraperitoneally with 1 mg BrdU (Santa Cruz, Heidelberg, Germany) in 100 μl PBS 2 h prior to sacrifice and organ harvest. Intracellular staining was performed using the anti-BrdU antibody (Abcam, Paris, France, 1/100 dilution) after acid treatment to unwind the DNA and help antibodies access to DNA incorporated BrdU. After neutralization with sodium borate, anti-BrdU Ab was revealed with goat anti-rat Alexa fluor 488 (Abcam, Paris, France, 1/500 dilution). Macrophages were then stained with an anti-CD68 Ab (Biolegend, Saint-Quentin-En-Yveline, France, 1/200 dilution) revealed with goat anti-rat Alexa fluor 555 (Abcam, Paris, France, 1/500 dilution). DAPI- and WGA-stained nuclei and membranes, respectively.

### Isolation and Preparation of immune cells for flow cytometry analysis

After perfusion with PBS, the mice heart was excised and digested in HBSS medium containing 2.5 mg/ml collagenase D (Roche, Meylan, France) for 30 min at 37 °C, with stirring.

Erythrocytes were lysed by using red blood cell lysis buffer (MiltenyiBiotec, Paris, France). Samples were blocked with Fc block (Ebioscience, Paris, France) prior to labeling with antibodies and propidium iodide (PI). Cytometry data were acquired on an LSR Fortessa cytometer. After gating on CD45^+^ cells, doublets were excluded and live cells were analyzed (PI exclusion). Cardiac cell numbers were quantified using polybeads (Polysciences, Le-Perray-en-Yvelines, France). Data were analyzed with FlowJo software (Tree Star).

### Preparation of immune cells for fluorescence activated cell sorting

Cardiac immune cells were isolated by centrifugation, enriched by immunoselection using an anti-CD45 antibody coupled to magnetic beads (MiltenyiBiotec, Paris, France). Samples were blocked with Fc block (Ebioscience, Paris, France) prior to labeling with antibodies. Cytometry data were acquired on a BD FACSAria II cell sorter (5 lasers). After gating on CD11b^+^ cells, doublets were excluded and live (PI exclusion) CD14^+^/CD64^+^ Mφ were sorted directly into RLT lysis buffer (Qiagen) and kept at −80 °C until RNAseq analysis.

Antibodies/flow cytometry/sorting/western blotting.

**Table:** 

Experiment	Target	Clone	Isotype	Reference	Dilution	Fluorochrome	Source
Analysis	CD11b	REA-592	Human IgG1	130-109-290	1: 80	Vio Bright FITC	MiltenyiBiotec Paris, France
Analysis	CD45	30-F11	Rat IgG2b, κ	48-0451-82	1: 200	eFluor 450	Ebioscience Paris, France
Analysis & sorting	CD64	REA-286	Human IgG1	130-103-808	1: 40	PE	MiltenyiBiotec Paris, France
Analysis & sorting	MHC-II	M5/114.15.2	Rat IgG2b, κ	56-5321-82	1: 400	Alexa Fluor 700	Ebioscience Paris, France
Analysis	CCR2	475301	Rat IgG2b	FAB5538A	1: 10	APC	R&D Systems Abingdon UK
Analysis	Ly6C	AL-21	Rat IgM, κ	560596	1: 400	APC-Cy 7	BDBiosciences Le-Pont-De-Claix, France
Sorting	CD11b	M 1/70	Rat IgG2b, κ	48-0112-82	1: 200	eFluor 450	Ebiosciences Paris, France
Sorting	CD14	SA2-8	Rat IgG2a, κ	11-0141-82	1: 200	FITC	Ebiosciences Paris, France
WB	CX3CL1	polyclonal	Rabbit IgG	TP233	1: 1000		Torrey Pines Secaucus USA
WB	CX3CR1	polyclonal	Rabbit IgG	Ab8021	1: 1000		Abcam, Paris, France
WB	TNFα	EPR20972	Rabbit	Ab215188	1: 1000		Abcam, Paris, France
WB	GAPDH	14C10	Rabbit	2118	1:25000		Cell signal, St-Cyr, France

### RNA sequencing and statistical analysis

Total RNA from FACS sorted CD64^+^/CD14^+^ cells was isolated using the Nucleospin RNA XS kit (Macherey Nagel, Hoerdt, France), according to the manufacturer instructions. cDNA libraries were generated using total RNA with SMART-Seq v4 Ultra Low Input RNA Kit (TAKARA) and constructed according to manufacturer protocols as previously reported^18^. Paired end sequencing (2 × 750 bp) was performed by Nextseq 500 machine using High Output kit (150 cycles). Raw sequencing data was quality-controlled with the FastQC program. Trimmomatic was used to remove adapter sequences, trim low-quality reads, and discard reads shorter than 40 bp. Reads were aligned to the mouse reference genome (build mm10) with the TopHat2 tool. Mapping results were quality-checked using RNA-SeQC. Aligned reads were counted using the FeatureCounts and Express software, at the gene-level and transcript-level, respectively. Normalization and differential analysis were performed with the GLM EdgeR package. RNA-Seq data has been made publicly available through the NCBI Gene Expression Omnibus (GEO), GEO accession number GSE157035.

### Quantitative RT-PCR

Quantitative RT-PCR experiments are detailed in supplementary material.

### Immunofluorescence

Immunofluorescence studies were performed on frozen sections or cells fixed in paraformaldehyde as described in^15^.

Tissue sections and cells were analyzed with a Zeiss Axio Observer Z1 microscope. Image analysis was performed using ImageJ and Photoshop CS5 (Adobe, San Jose, CA, USA). Results are expressed as the number of positive cells per field and were quantified from 4–10 mice per group and 20–32 images per animal.

### Quantification of cardiomyocyte area and tissue fibrosis

Frozen sections fixed in paraformaldehyde were labeled with WGA-Alexa 647 (Thermo-Scientific, Montigny-Le-Bretonneux, France, 1/500 dilution). Tissue sections were analyzed with a Zeiss Axio Observer Z1 microscope using ImageJ software. A low *vs.* high threshold allowed quantification of cardiomyocyte area or tissue fibrosis, respectively, as previously reported^15^. Results were quantified from 6–7 mice/group (12–32 images/animal).

### Western blot

Isolated cardiomyocytes or tissue homogenates were lysed in 150 mM NaCl, 50 mM Tris pH = 7.4, EDTA 1 mM, EGTA 1 mM, Na_4_P_2_O_7_ 5 mM, 10% glycerol, 1% triton, and protease and phosphatase inhibitors cocktail (Sigma, St-Quentin-Fallavier, France). Samples were then centrifuged at 3000 × *g* for 5 min to get rid of cell debris. Protein concentration was measured by the BCA Protein assay (Thermo-Scientific, Montigny-Le-Bretonneux, France).

Proteins were separated on NuPAGE Novex 10% or 4–12% Bis–Tris gels (Life Technologies) and transferred on nitrocellulose membrane (Biorad, Marnes-La-Coquette, France). Membranes were cut and each part was incubated with indicated antibodies. Incubations were performed with appropriate primary antibodies (see antibodies listing above) followed by HRP-coupled secondary antibodies (Cell Signal, St-Cyr, France). Proteins were revealed with ECL Prime (GE Healthcare, Velizy, France) and images were acquired using a LAS4000 Camera (GE Healthcare, Velizy, France).

We displayed cropped gels and blots in the main paper to improve the clarity and conciseness of the presentation. However, full-length unedited material was provided in the supplementary information, as mentioned in the figure legends.

### Drugs

Sources of drugs are detailed in supplementary material.

### Statistical analysis

Quantitative data are reported as means ± SEM. Statistical analysis was performed with GraphPad Prism 8 (GraphPad software Inc, San Diego, CA, USA). For multiple comparisons of values D'agostino-and-Pearson normality test was first performed, and Kruskal–Wallis test, one-way ANOVA or 2-way ANOVA were used as appropriate followed by a post-hoc test for pairwise multiple comparisons. Correlation studies were performed using the Spearman correlation coefficient. Survival analysis was performed using the Gehan-Breslow-Wilcoxon test. All values with p < 0.05 were considered significant.

## Results

### β-adrenergic stimulation induces a transient early concentric hypertrophy (ECH) remodeling associated with an emergence of cardiac Mφ

**Figure 1:**
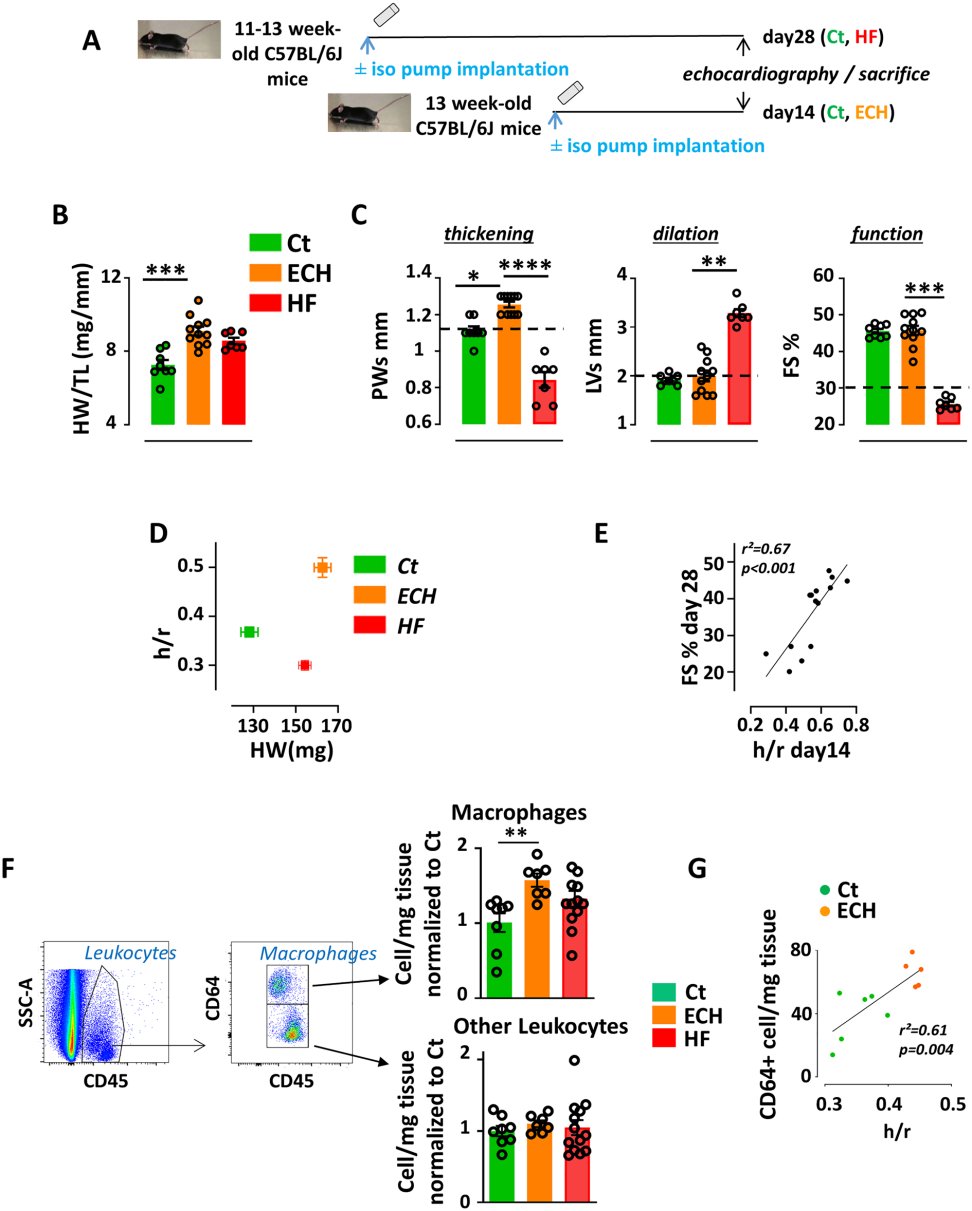
An increase in CD64^+^ Mφ is correlated with concentric hypertrophy in iso-induced ECH hearts. Mice implanted or not with an iso-pump for 14 or 28 days were subjected to echocardiographic analyses. (**A**) Schematic representation of the protocol. (**B**) Heart weight/tibia length; n = 8 (Ct), 11 (ECH) and 7 (HF) mice, Kruskal–Wallis followed by Dunn’s post-hoc test; *** p < 0.001. (**C**) Echocardiographic parameters; n = 8 (Ct), 11 (ECH) and 7 (HF) mice, Kruskal–Wallis followed by Dunn’s post-hoc test; *p < 0.05, **p < 0.01, ***p < 0.001, ****p < 0.0001. (**D**) From same mice, evolution of the geometric parameter h/r (diastolic wall thickness to radius ratio) and heart weight. (**E**) Mice implanted with an iso-pump for 28 days: Spearman correlation coefficient between h/r at day 14 and FS at day 28; n = 13 from 2 different protocols. (**F**) Cardiac cells isolated from mice implanted or not with an iso-pump for 14 or 28 days were analyzed by flow cytometry. Cells were isolated from collagenase digested hearts, stained with the indicated antibodies and submitted to typical flow cytometry gating strategy to identify cardiac CD64^+^ Mφ. CD45^+^ leukocytes were identified, doublets excluded (by FSC-W vs. SSCA). Live CD45^+^ cells (after PI exclusion) were gated on CD64^+^ Mφ. Quantification of immune cells; n = 8 (Ct), 7 (ECH) and 13 (HF) cell isolation, Kruskal–Wallis followed by Dunn’s post-hoc test; ** p < 0.01. (**G**) Spearman correlation coefficient between cardiac CD64^+^ (flow cytometry analysis) cell number/mg and h/r; n = 6 mice (Ct), n = 5 mice (ECH).

As recently reported^15^, cardiac remodeling was induced in 11–13 week-old male C57BL/6J mice by chronic isoproterenol stimulation (30 mg/kg/day) for 14 days or 28 days (Fig. 1A).

At day 14, mice developed compensatory cardiac hypertrophy with increased ratio of heart weight/tibia length (HW/TL) and thickening of posterior wall (PWs) and interventricular septum (IVSd and IVSs) but preserved systolic function (EF and FS) (Fig. 1B,C and Table S1). This early hypertrophic response displayed a concentric pattern evidenced by a combined increase in the geometric parameter h/r (diastolic wall thickness to radius ratio) with the heart weight (HW) (Fig. 1D).

At day 28, the iso-infused mice displayed an evolution towards heart failure (HF) characterized by left ventricular (LV) dilation (LVd and LVs) associated with lower PWs, PWd, IVSs and IVSd and alteration of the cardiac function (EF and FS) (Fig. 1C and Table S1). Although HW/TL ratio indicated a persistent hypertrophy in hearts (Fig. 1B), the combination of a high HW with a low h/r as compared to control mice evidenced a progression towards an eccentric geometry (Fig. 1D). HF hearts also displayed higher fibrosis as compared to control hearts (Figure S1).

The protective role of the initial early concentric hypertrophy (ECH) remodeling against the alteration of function was suggested by a positive correlation between the intensity of the concentric hypertrophy (h/r) at day 14 and the preservation of FS at day 28 (Fig. 1E).

Flow cytometry analysis of CD45^+^ leukocytes in control (Ct), ECH and HF hearts showed that CD64^+^ Mφ (a pan-Mφ surface marker) were transiently increased in ECH hearts with no statistical change in the number of other leukocytes (Fig. 1F). Of note, the CD64^+^ Mφ cell number was correlated with the concentric hypertrophy level (h/r parameter) (Fig. 1G). A correlation between the h/r parameter and the cardiac CD68^+^ cell number (a pan-Mφ cytoplasmic marker) measured by immuno-histological tissue staining was also observed (Figure S2A).

### Cardiac myeloid cells from ECH hearts selectively exert a pro-hypertrophic effect in-vitro and clodronate-induced depletion hampers the β-adrenergic-induced early hypertrophic response

**Figure 2:**
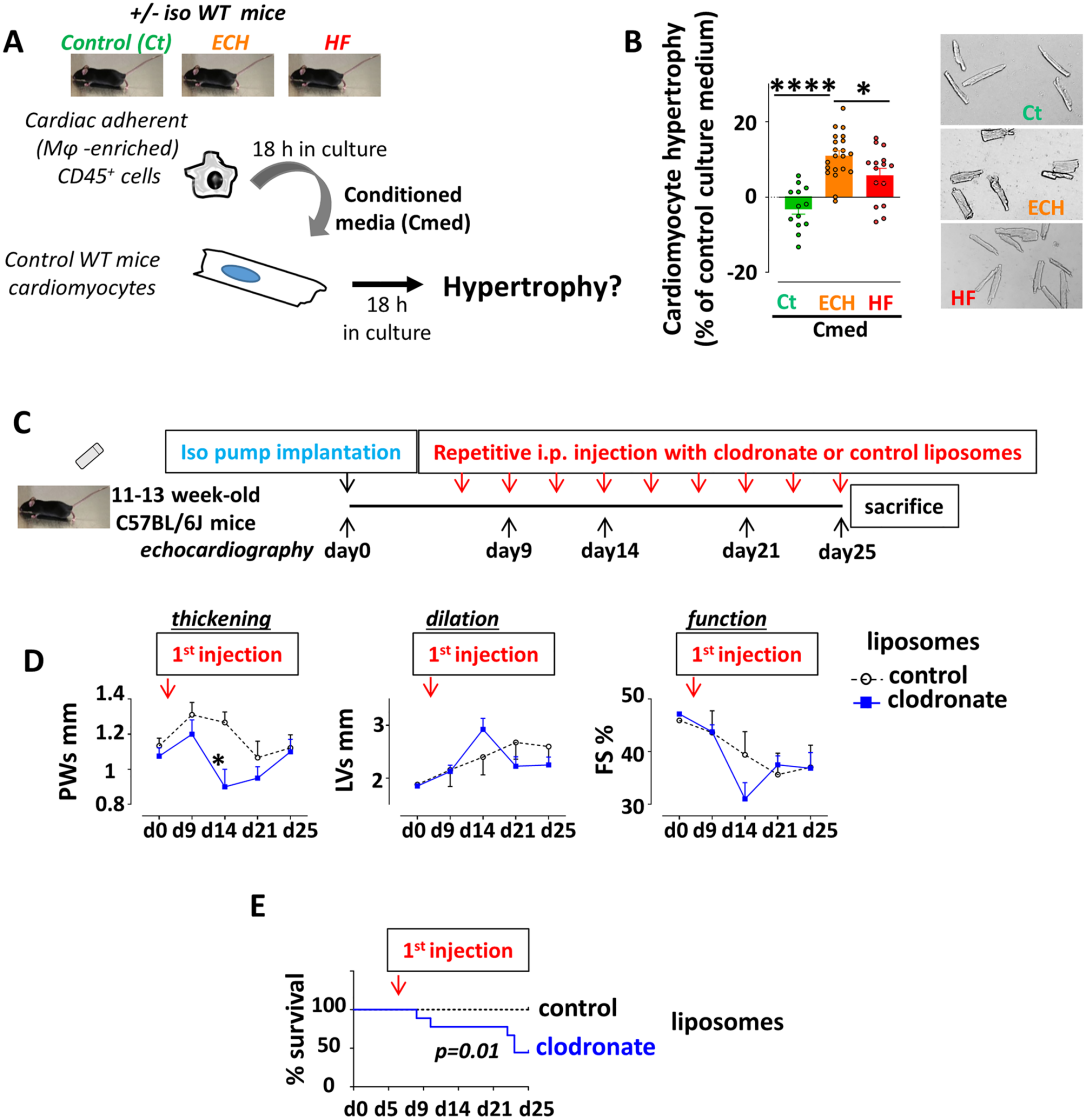
Mφ-enriched adherent CD45^+^ cells from ECH hearts selectively trigger adult WT cardiomyocyte hypertrophy in-vitro. Mφ depletion during ECH hampers early hypertrophy in-vivo. (**A**) Experimental procedure. Cardiac immune cells were isolated from collagenase digested Ct, ECH and HF hearts. Following CD45 positive enrichment using mouse CD45 microbeads, adherent (Mφ-enriched) cells were kept in culture for 18 h before conditioned media (Cmed) recovery. Cmed were applied on cardiomyocytes from WT mice. Cell hypertrophy was analyzed 18 h later. (**B**) Cmed from adherent CD45^+^ isolated from iso-infused ECH WT mice selectively enhance hypertrophy of control WT cardiomyocytes. Mean ± SEM of 5 experiments performed in triplicate. Cardiomyocytes from 5 mice (225–750 cell area quantified per condition per experiment), Cmed from 4–10 mice, Kruskal–Wallis followed by Dunn post-hoc test; * p < 0.05, **** p < 0.0001. (**C**) Experimental procedure of clodronate or control liposomes treatment in mice implanted with an iso-pump. (**D**) Echocardiographic parameters measured at d0, d9, d14, d21 and d25; n = 4–9 mice/group, two-way ANOVA followed by Sidak’s post-tests; * p < 0.05 vs control at the same time point. (**E**) Survival of control or clodronate liposomes treated mice; n = 9 mice/group at d0, Gehan-Breslow-Wilcoxon Test.

In order to assess the direct selective impact of ECH myeloid cells on adult cardiomyocyte hypertrophy, we immunoselected CD45^+^ leukocytes from control, ECH and HF hearts (iso-infused mice) and only kept adherent (non-lymphocyte) CD45^+^ cells comprising an averaged 64.2 ± 3.5% CD64^+^ cell number (n = 10). We collected the conditioned medium (Cmed) after 18 h in culture, before challenging isolated adult cardiomyocytes with the different Cmed and evaluating their mean area 18 h later (Fig. 2A).

Only ECH Cmed triggered a significant increase in cardiomyocyte area, as compared to control and HF Cmed, with a mean 11 ± 1.3% increase in cell area (n = 21) (Fig. 2B), characterized by a selective increase in the cell width but no change in cell length (Figure S3A). Unfortunately, our attempts to reproduce this protocol using the Cmed collected from CD64^+^ cells sorted by flow cytometry failed, likely because of the limited cell yield leading to a too low concentration of Cmed.

To evaluate the physiological impact of myeloid cells in ECH hearts, the iso-infused mice were subjected to repetitive i.p. injections of clodronate or control liposomes (100 µl/25 g) three times a week beginning at day 7 (during ECH) following pump implantation and until sacrifice (Fig. 2C). Clodronate liposomes-treated mice displayed a hampered compensatory hypertrophic response with significantly lower PWs at day 14 and decreased PWd at day 9 and 14 as compared to control liposomes-treated mice (Fig. 2D and Table S2). In addition, their cardiac function (FS) tended to be more altered at day 14 (Fig. 2D) and their survival was significantly affected (Fig. 2E). As expected, the clodronate liposomes-treated mice displayed a decrease in cardiac CD68^+^ cell number attesting for efficient clodronate-induced myeloid cell depletion (Figure S2B). Of note, clodronate neither promoted apoptosis nor amplified iso-induced fibrosis (Figure S2B).

Taken together, these results highlighted an increase in Mφ number during the iso-induced ECH remodeling. Mφ-enriched cardiac adherent CD45^+^ cells from ECH hearts directly induced concentric hypertrophy of cardiomyocytes and the myeloid cell-depleting clodronate treatment neutralized the ECH remodeling, arguing for the potential in-vivo transient protective role of Mφ in iso-induced ECH.

### ECH Mφ are characterized by enhanced anti-inflammatory-, phagocytic- and chemotactic-related gene expression, with *Cx3cl1* as the most upregulated gene coding for a secreted factor

**Figure 3:**
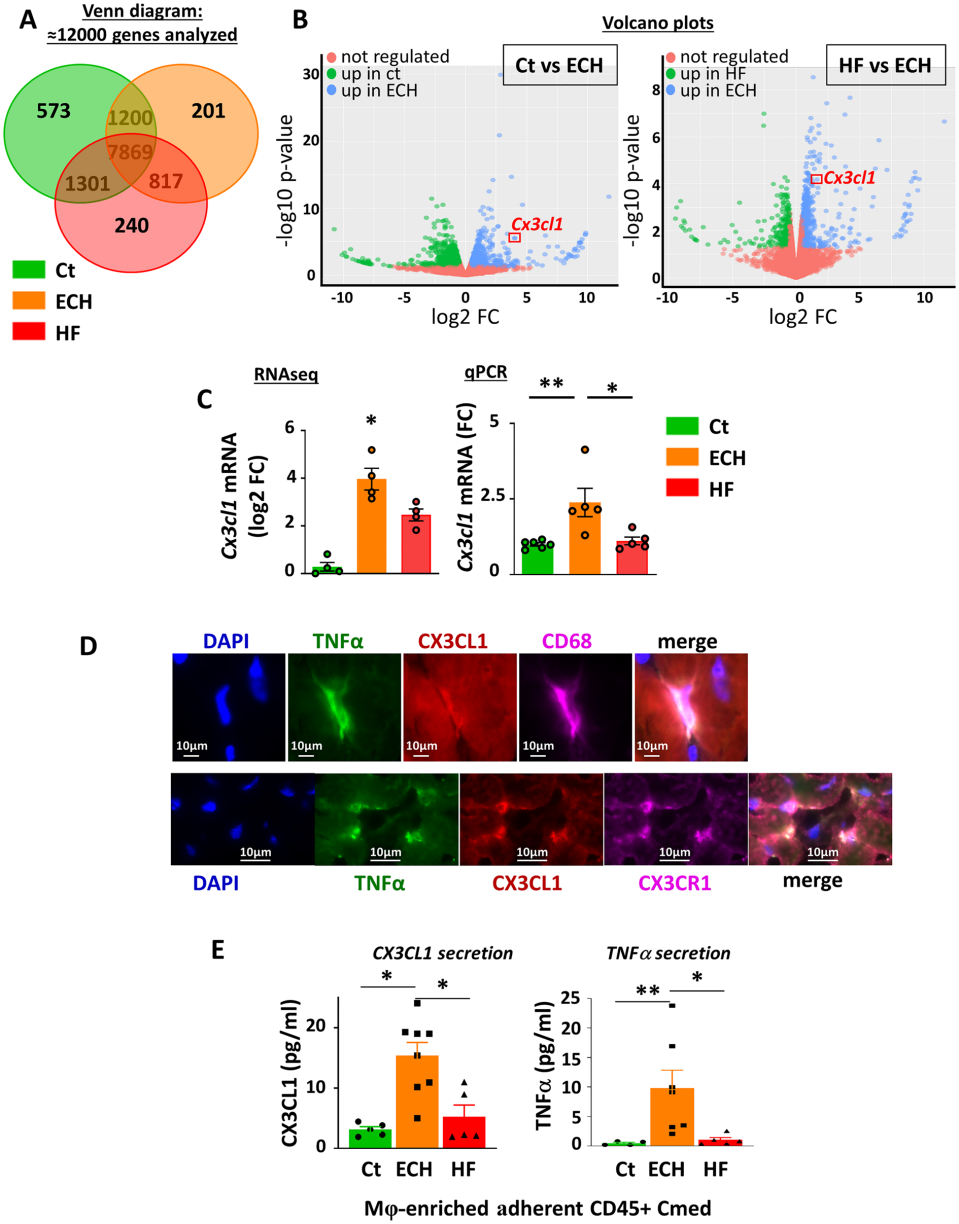
Cardiac ECH CD64^+^ Mφ are characterized by a typical induction of *Cx3cl1* mRNA and a co-secretion of CX3CL1 and TNFα, as compared to Ct and HF counterparts. (**A**) RNAseq transcriptomic analysis: three-way Venn diagrams. (**B**) Volcano Plots showing the number of genes differentially expressed between ECH and Ct or ECH and HF CD64^+^ Mφ***. ***(**C**) Analysis of *Cx3cl1* mRNA levels in isolated cardiac CD64^+^ cells by RNAseq (n = 4 mice/group, normalization and differential analysis were performed with the glm edgeR package) and qPCR (n = 6 (Ct), 5 (ECH) and 5 (HF) mice, Kruskal–Wallis followed by Dunn post-hoc test; * p < 0.05, ** p < 0.01). (**D**) Typical immuno-fluorescent stainings of cardiac sections from iso-infused ECH mice with anti-TNFα, CX3CL1, CD68 or CX3CR1 Abs. (**E**) Multiplex CX3CL1 and TNFα analysis in Cmed from adherent Mφ***-***enriched CD45^+^ cells prepared as described in Fig. 2A; n = 5 (Ct), 8 (ECH) and 5 (HF) mice, Kruskal–Wallis followed by Dunn’s post-hoc test; * p < 0.05, ** p < 0.01.

We used RNA sequencing (RNAseq) to characterize the ECH Mφ as compared to control and HF Mφ and to highlight mechanisms by which they might mediate their protective function. Following CD45 positive enrichment, we sorted the cardiac Mφ using two well characterized markers, CD64 and CD14, according to the gating strategy described in Figure S4. Control, ECH and HF sorted cells expressed homogenous levels of myeloid markers such as *Cd14*, *Csf1r* or *Adgre1* and *Fcgr1* coding for F4/80 and CD64, respectively. However, RNAseq clearly indicated a typical transcriptional program in ECH Mφ, since differential gene expression analysis revealed 201 genes that were selectively regulated in ECH Mφ as compared to control and HF Mφ (using a threshold of twofold change and false discovery rate (FDR) < 0.05) (Fig. 3A).

Volcano plots showed the number of genes differentially expressed between control Mφ vs ECH Mφ (Fig. 3B left panel) and HF Mφ vs ECH Mφ (Fig. 3B right panel).

ECH Mφ were characterized by up-regulated *Mertk* (phagocytic marker) gene expression (Figure S5B and S5 C). In keeping with *Mertk* gene induction, immuno-histological analyses confirmed a selective transient increase in CD64^+^/Mer^+^ and CD68^+^/Mer^+^ Mφ in ECH hearts (Figure S6A and S6B) that were depleted upon clodronate treatment (Figure S6C and S6D). ECH Mφ also displayed up-regulated *Il4R*a, *Arg1* and *Cd163* (M2 markers) gene expression (Figure S5C) and a downregulation of *Cd209* dendritic cell markers (Figure S5B). ECH Mφ also showed downregulated *Mhc-2* (*H2-aa* and *H2-ab1*) expression as compared to control (Figure S5D). In addition, ingenuity pathway analysis (IPA) demonstrated that interferon gamma (IFNγ) was the top upregulated signaling pathway identified by upstream analysis (z-score 2.508 and p-value 1.06 E^−3^).

IPA indicated that the genes selectively upregulated in ECH Mφ were involved in Th2 pathways, quantity of connective tissue (inhibition of MMPs), phagosome formation (phagocytosis) and chemotactism (Figure S5A).

Importantly, analyses of both inflammatory cytokines and chemokines gene expression patterns in ECH Mφ identified *Cx3cl1* coding for CX3CL1 among the top upregulated genes (Fig. 3B,C left panel and S5B). Of note, there was no change detected in the expression of its unique receptor *Cx3cr1* (not shown). Selective transient induction of *Cx3cl1* in ECH CD64^+^ Mφ was confirmed by qPCR (Fig. 3C right panel).

Immuno-fluorescent co-staining of cardiac tissues from iso-infused ECH mice suggested that CD68^+^ or CX3CR1^+^ Mφ were able to produce both TNFα and CX3CL1 (Fig. 3D). This was in keeping with the upregulation of *Adam8* and *Adam10* gene expression detected in ECH Mφ (Figure S5E) since ADAM proteins are known to mediate cleavage of surface molecules among which CX3CL1 and TNFα^19^. A multiplex analysis of secreted molecules detected in the Cmed from Mφ-enriched adherent CD45^+^ cells isolated from Ct, ECH and HF hearts confirmed a selective increased release of CX3CL1 by ECH cells and showed an associated secretion of TNFα (Fig. 3E). Our next experiments aimed to evaluate the impact of the CX3CL1/CX3CR1 axis on ECH Mφ-enriched adherent CD45^+^ cells emergence and cardiomyocyte-related hypertrophy.

### The CX3CL1/CX3CR1 axis drives a direct pro-hypertrophic effect of ECH Mφ-enriched CD45^+^ cells and favors Mφ proliferation and expansion

**Figure 4:**
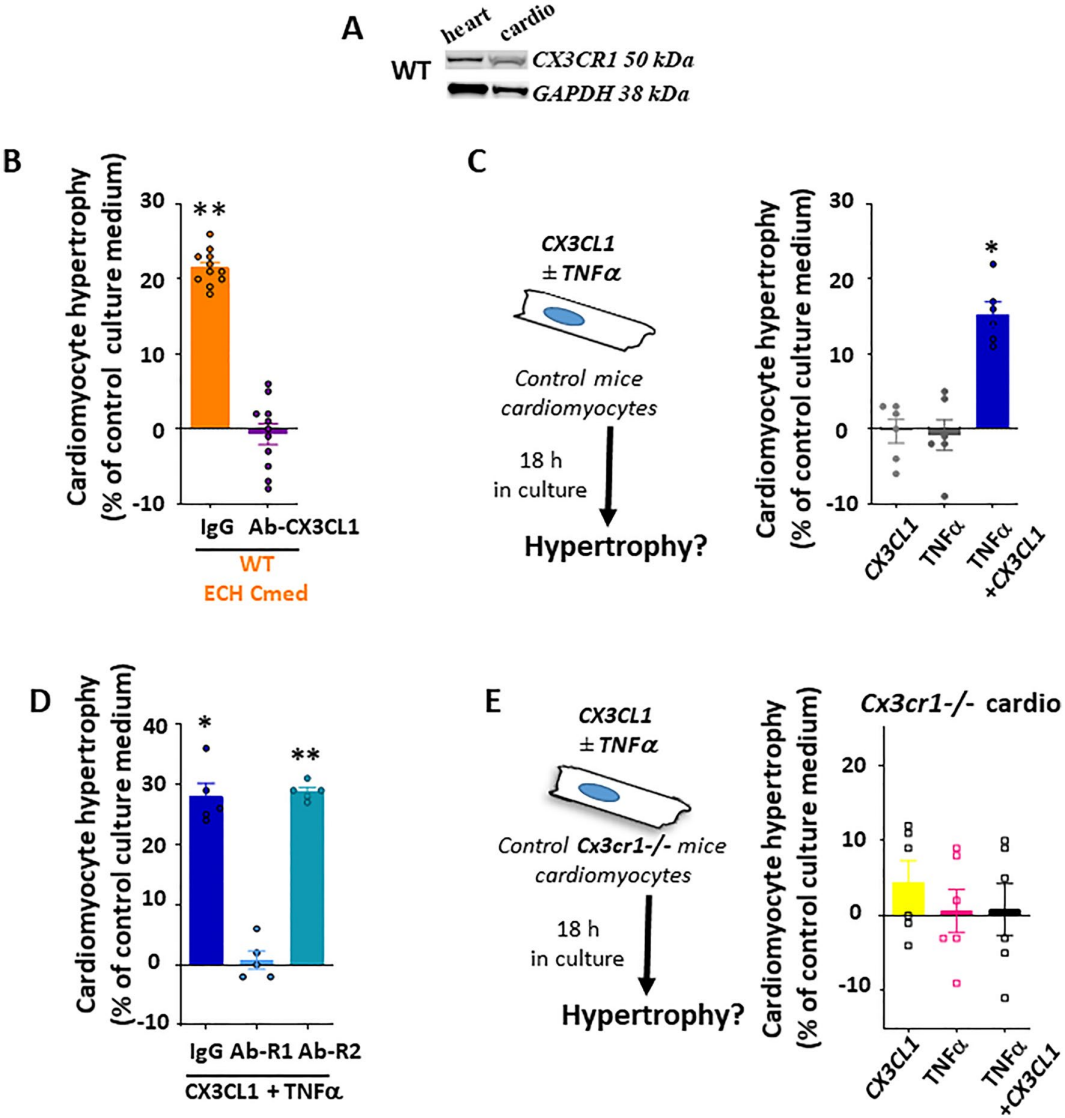
Determinant role of the CX3Cl1/CX3CR1 axis in the pro-hypertrophic effect of ECH Mφ-enriched CD45^+^ cells in adult WT cardiomyocytes: synergism between CX3CL1 and TNFα. (**A**) CX3CR1 protein expression in WT heart tissue (15 µg) or isolated adult WT cardiomyocytes (50 µg). Full unedited gels are provided in the supplementary material as well as detailed procedure (Figure S10). (**B**) Cmed from adherent CD45^+^ cells isolated from ECH WT mice enhances hypertrophy of WT cardiomyocytes in a CX3CL1-dependent manner. Experimental procedure described in Fig. 2A. (**C**) CX3CL1 requires the synergistic action of TNFα to enhance hypertrophy in control WT cardiomyocytes. (**D**) The pro-hypertrophic impact of CX3CL1 + TNFα relies on TNFR1 binding of TNFα. (**E**) Either alone or in combination, CX3CL1 and TNFα are without hypertrophic effect on adult cardiomyocytes from control *Cx3cr1*^*-/-*^ mice. Mean ± SEM of 2–6 experiments performed in triplicate. Cardiomyocytes from 2–6 mice (225–750 cell area quantified per condition per experiment), Cmed from 4–10 mice, Kruskal–Wallis followed by Dunn post-hoc test; * p < 0.05, ** p < 0.01 vs Ct medium.

Adult cardiomyocytes expressed CX3CR1, the unique CX3CL1 receptor (Fig. 4A).

Treatment with neutralizing CX3CL1-antibody blunted the pro-hypertrophic effect of the Cmed collected from ECH Mφ-enriched CD45^+^ cells (hypertrophy decreased from 21.5 ± 0.7% to − 0.7 ± 1.4%, n = 11) (Fig. 4B). Interestingly, CX3CL1 alone did not induce cardiomyocyte hypertrophy (tested either at a current 50 ng/ml concentration (-0.3 ± 1.6% hypertrophy, n = 6) (Fig. 4C) or at a 100 ng/ml higher dose (not shown)). However, ECH myeloid cells secreted not only CX3CL1 but also TNFα (Fig. 3D,E). Since CX3CL1 and TNFα have been reported to potentially act in a synergistic manner^20,21^, we tested the hypothesis of a combined CX3CL1 and TNFα effect in the pro-hypertrophic action of ECH Cmed. Alone, 50 ng/ml TNFα did not induce cardiomyocyte hypertrophy (− 0.8 ± 2% hypertrophy, n = 6), in contrast to the combination of 50 ng/ml CX3CL1 and 50 ng/ml TNFα (15.3 ± 1.6% increase in cell area, n = 6) (Fig. 4C). This hypertrophy was characterized as concentric with a selective impact on cell width but no change in cell length (Figure S3B). Similar experiments performed on adult cardiomyocytes isolated from *Cx3cr1*^*-/-*^ mice^16^ showed no hypertrophic effect of CX3CL1 and TNFα either alone or in combination (Fig. 4E), highlighting the crucial role of CX3CR1 expression in hypertrophic remodeling (Fig. 4A). The combined CX3CL1 and TNFα pro-hypertrophic effect depended on the binding to TNFR1 but not to TNFR2 (Fig. 4D).

The CX3CL1/CX3CR1 axis is known as a pro-survival pathway in Mφ^22,23^. In line, in-vitro experiments performed in Mφ-enriched CD45^+^ cells isolated from iso-infused ECH mice, showed that CX3CL1 increased the number and the proliferative activity of CD68^+^ cells (Ki67 staining, Figure S7A). Of note, CX3CL1 proliferative impact was as potent as that elicited by the mitogenic agent M-CSF (50 ng/mL) and was not amplified upon addition of TNFα (50 ng/mL) (not shown). Furthermore, an in-vivo CD68^+^ cell proliferation was detected in hearts infused with isoproterenol for 8 days and injected with BrdU 2 h before sacrifice with an enhanced number of CD68^+^ Mφ positive for BrdU as compared to control hearts (35.6 ± 2.8% vs 16.4 ± 2.7% of CD68^+^ Mφ were BrdU^+^ in iso-infused vs control hearts, respectively; n = 6 vs 5 mice, p < 0.01) (Figure S7B).

Altogether, these data suggested a direct role of the CX3CL1/CX3CR1 axis on the expansion of cardiac Mφ and their pro-hypertrophic effect on adult cardiomyocytes.

### Protective role of the CX3CL1/CX3CR1 axis during iso-induced ECH remodeling

**Figure 5:**
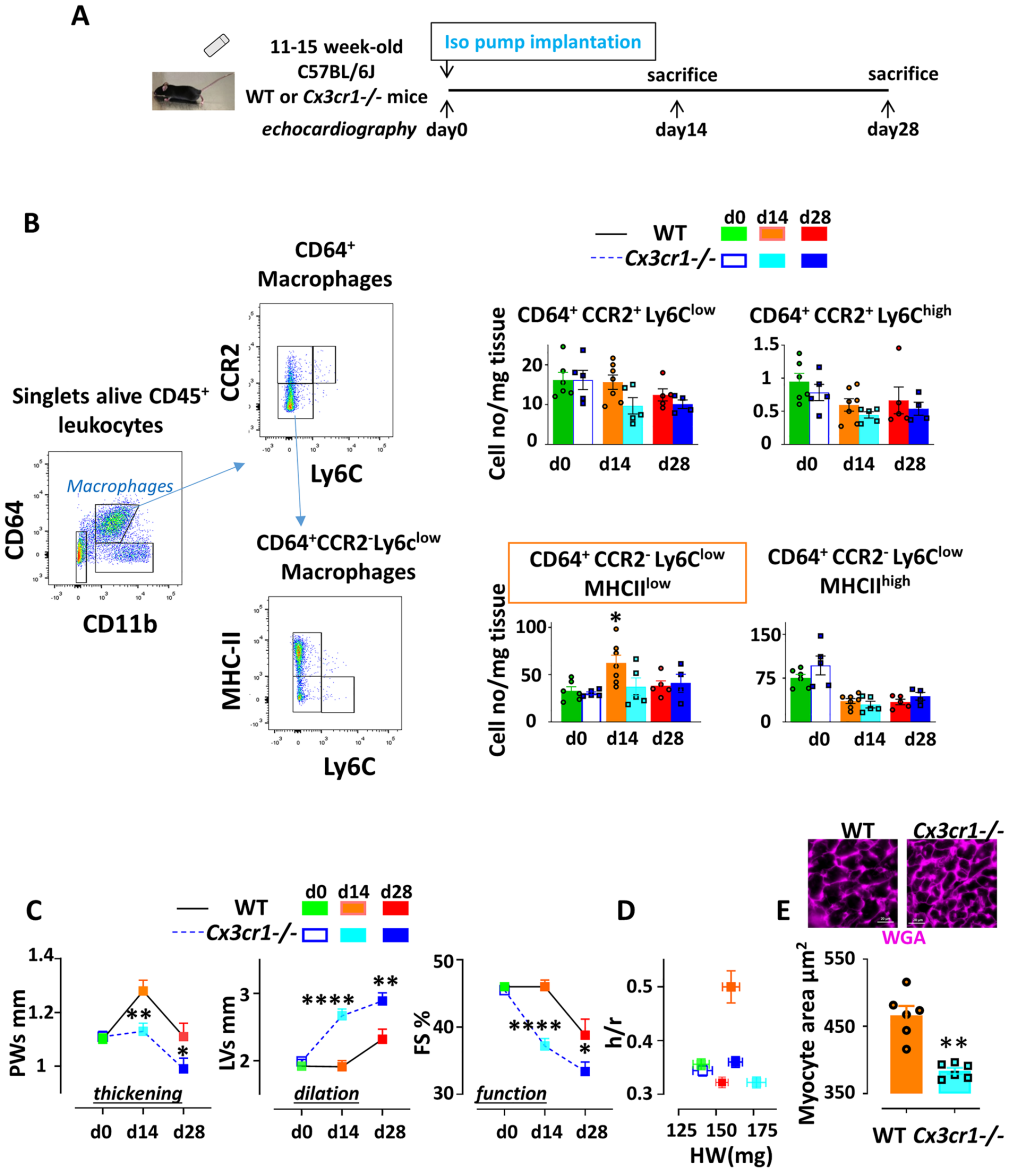
*Cx3cr1* knockout hampers the transient selective increase in CD64^+^ CCR2^-^ Ly6C^low^ MHCII^low^ Mφ detected in WT mice during ECH, suppresses iso-induced concentric hypertrophy and accelerates dilation and alteration of function. (**A**) Experimental procedure (**B**) Typical flow cytometry gating strategy to identify cardiac Mφ subpopulations. Cells were isolated from collagenase digested hearts and stained with the indicated antibodies before analysis. CD45^+^ leukocytes were identified, doublets excluded (by FSC-W vs. SSCA). Live CD45^+^ cells (after PI exclusion) were gated on CD11b^+^ CD64^+^ Mφ. CD11b^+^ CD64^+^ Mφ were further analyzed on their pattern of expression of CCR2 vs Ly6C and CCR2^-^ Ly6C^low^ cells were gated on MHCII^high^ and MHCII^low^ cells. Quantification of immune cells n = 4–7 cell isolation, two-way ANOVA followed by Sidak’s post-hoc tests. * p < 0.05 vs WT at the same time point. (**C**) Kinetics of echocardiography parameters in WT or *Cx3cr1*^*-/-*^ mice implanted with Iso pump at day 0. n = 14–38 mice, Two-way ANOVA followed by Sidak’s post-hoc tests. * p < 0.05, ** p < 0.01, *** p < 0.001, **** p < 0.0001 vs WT at the same time point. (**D**) Geometric parameter h/r versus HW n = 7–26 mice/group. (**E**) Cardiomyocyte cross-sectional area estimated in cardiac sections stained with WGA (typical image). Mean ± SEM from 6 mice/group, sacrificed at day14. Mann–Whitney U test. ** *p* < 0.001.

We next examined, in-vivo, the impact of CX3CR1 knockout on Mφ evolution and cardiac remodeling in mice^24^ challenged with the iso-infusion (Fig. 5A).

As expected, flow cytometry analysis of Mφ showed a transient increase in CD64^+^ Mφ in ECH WT mice at day 14. On the basis of previously identified cardiac Mφ subpopulations^25^, we further characterized emerging ECH Mφ as CCR2^-^/Ly6c^low^/MHCII^low^ cells (Fig. 5B). In contrast, hearts from *Cx3cr1*^*-/-*^ mice did not exhibit any significant change in Mφ number in response to a 14-day iso-infusion (Fig. 5B). Importantly, *Cx3cr1*^*-/-*^ hearts displayed lower levels of both CX3CL1 and TNFα as compared to WT iso-induced ECH hearts (Figure S8A).

No significant echocardiographic differences were observed between WT and *Cx3cr1*^-/-^ mice at day 0 (Fig. 5C and Table S3). As expected, at day 14, WT mice exhibited typical ECH features such as an increase in PWs (Fig. 5C), h/r and HW (Fig. 5D), and no change in LVs and FS (Fig. 5C and Table S3). At day 28, WT mice began to evolve towards eccentric hypertrophy and HF with an increase in LVs and a decrease in FS (Fig. 5C). In contrast, iso-infused *Cx3cr1*^*-/-*^ mice lacked the early concentric response and directly exhibited eccentric remodeling (Fig. 5D) with a significant cardiac dilation and alteration of function at day 14 and a worsening at day 28 (Fig. 5C and Table S3). At day 14, *Cx3cr1*^*-/-*^ mice displayed a significant lower cardiomyocyte cross-sectional area as compared to WT mice (Fig. 5E).

Thus, neutralization of CX3CR1 in-vivo resulted in the lack of early expansion of Mφ and the absence of early concentric hypertrophy in mice infused with isoproterenol for 14 days. This was associated with a lack of in-vitro direct pro-hypertrophic impact of adherent CD45^+^ cells (not shown).

Taken together these results demonstrated the essential role of the CX3CL1/CX3CR1 axis in the early Mφ expansion and concentric hypertrophy remodeling elicited by iso-infusion in-vivo. At day 28 of iso-infusion, alteration of the CX3CL1/CX3CR1 pathway was associated with both increased apoptotic (cleaved caspase 3^+^ cells) and fibrotic (WGA staining) responses (Figure S8B and S8C).

**Figure 6:**
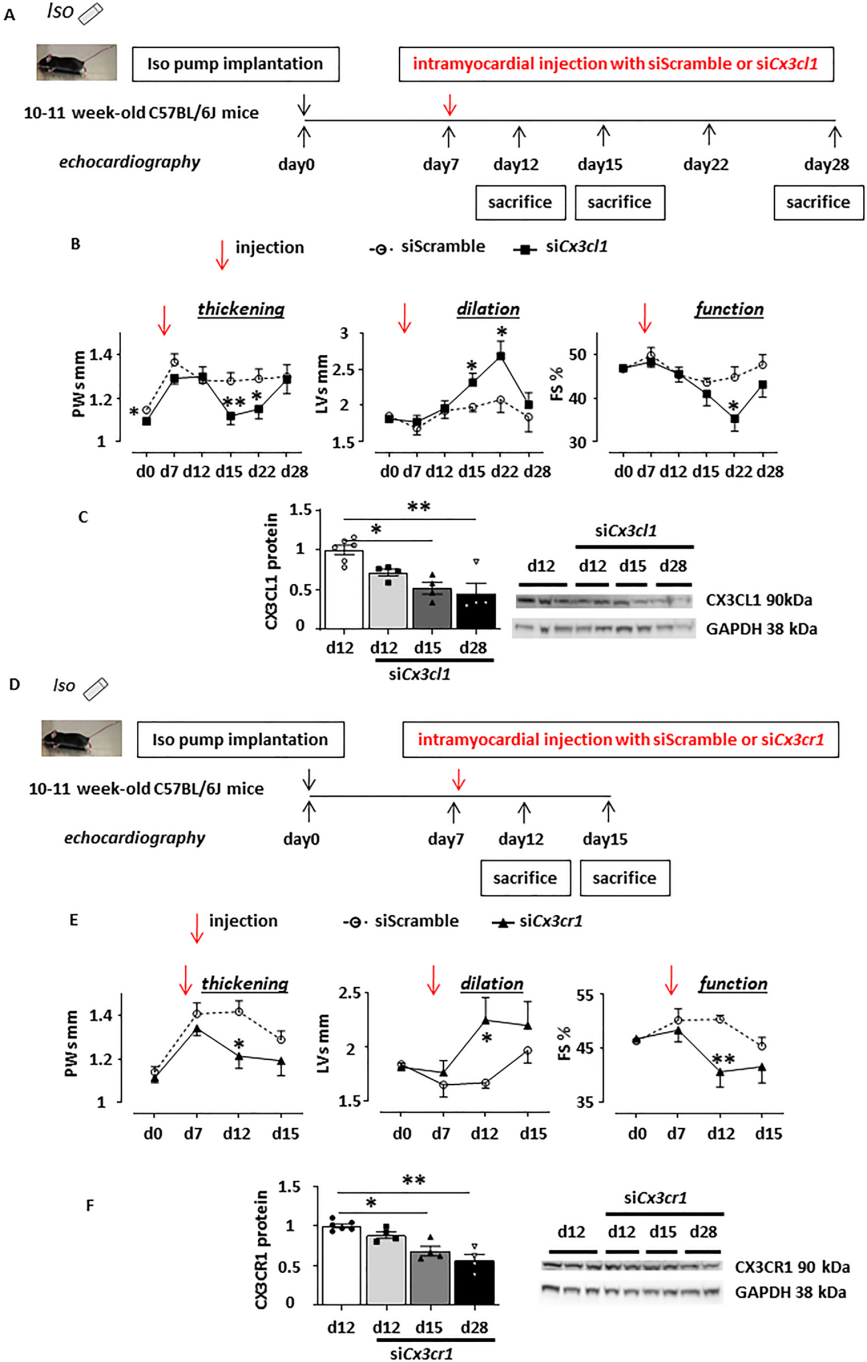
Cardiac CX3CL1 *or* CX3CR1 knockdown at the onset of ECH, via intramyocardial siRNA injection, reverses iso-induced concentric hypertrophy and favors alteration of function and dilation. (**A**,**D**) Schematic representation of the protocol where mice implanted with an iso-pump at day 0 were subjected to a unique ultrasound-guided intramyocardial transthoracic injection of Scramble, *Cx3cl1* or *Cx3cr1* siRNAs at day 7. (**B**,**E**) Echocardiographic parameters measured at d0, d7, d12, d15, d22 and d28 concerning siScramble or si*Cx3cl1* injections; n = 5–13 mice/group. Measurement at d0, d7, d12 and d15 for siScramble or si*Cx3cr1* injections; n = 10–14 mice/group, two-way ANOVA followed by Sidak’s post-hoc tests; * p < 0.05, ** p < 0.01 vs scramble at the same time point. (**C**,**F**) Efficient knockdown of CX3CL1 or CX3CR1 protein levels in cardiac homogenates at d12, d15 and d28 following siRNA injection. Full unedited gels are in the supplementary material as well as detailed procedure (Figure S12 and S13). Mean ± SEM of mice, n = 4–6 mice/group, Kruskal–Wallis followed by Dunn’s post-hoc tests; * p < 0.05, ** p < 0.01.

In order to strengthen the pathophysiological role of the cardiac CX3CL1/CX3CR1 axis in ECH, we next examined the impact of an in-vivo molecular knockdown at the onset of ECH. As previously reported^15^, iso-infused mice were subjected to a unique intramyocardial injection of either Scramble, *Cx3cl1* or *Cx3cr1* siRNA at day 7 following pump implantation (Fig. 6A,D and Figure S9).

Efficient knockdown of CX3CL1 or CX3CR1 during ECH was attested at the protein levels (Fig. 6C,F). At day 15 and day 22, hearts from si*Cx3cl1*-injected mice displayed a decreased ventricular thickening (lower PWs) and an increased dilation (higher LVs) and at day 22, an altered cardiac function (lower FS), as compared to hearts from siScramble-injected mice (Fig. 6B and Table S4). Mice injected with si*Cx3cr1* presented a similar lower thickening, an increased dilation, and an alteration of function at day 12 as compared to siScramble-injected animals (Fig. 6E and Table S5). Thus, CX3CL1 or CX3CR1 knockdown at the onset of ECH hampered cardiac concentric hypertrophy, accelerated dilation, and altered cardiac function. Taken together, these results demonstrated the emergence of a functional transient protective role of the cardiac CX3CL1/CX3CR1 axis playing during the critical temporal window of ECH remodeling and delaying evolution towards HF.

## Discussion

**Figure 7:**
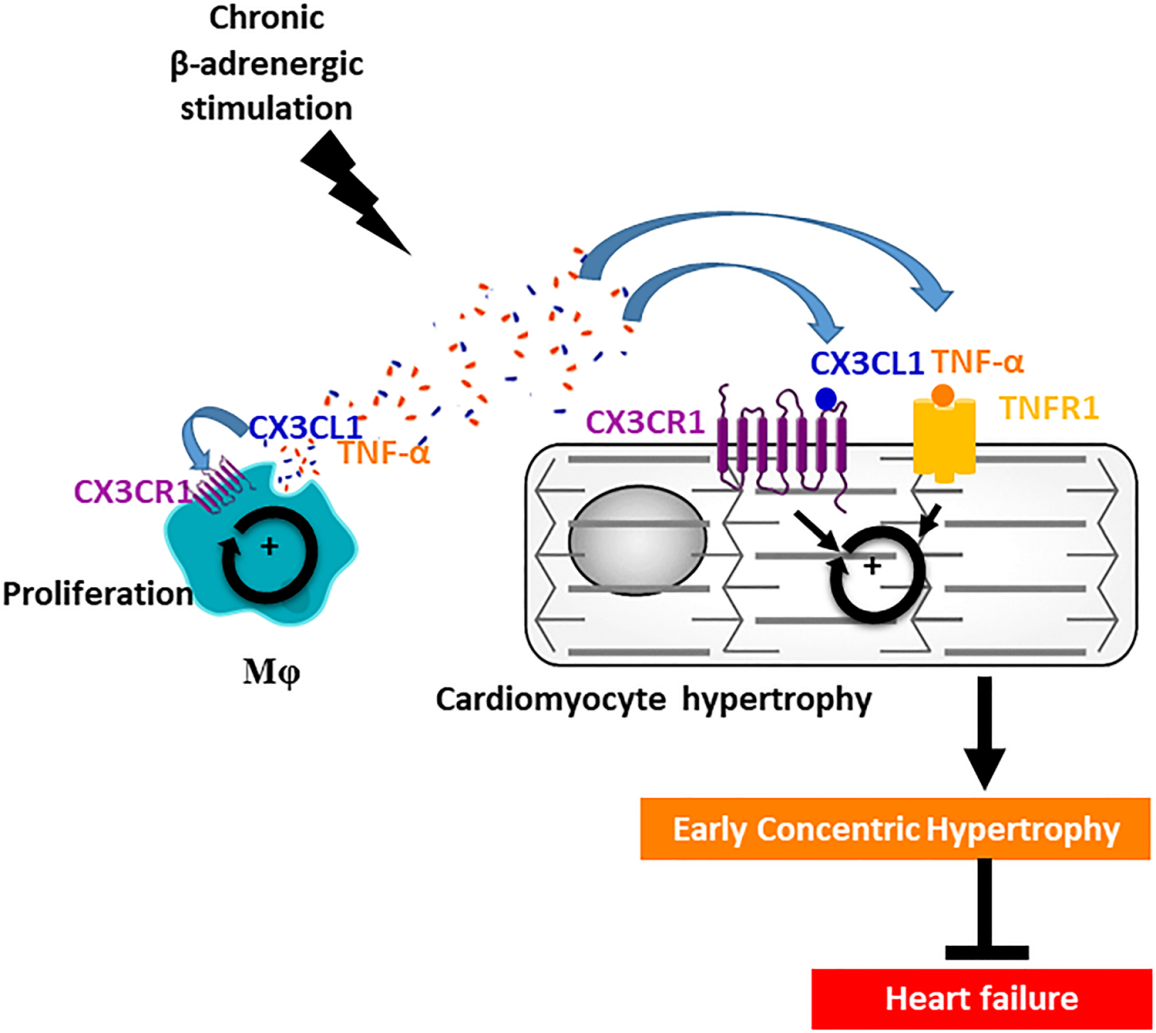
Protective role of the cardiac CX3CL1/CX3CR1 axis during β-adrenergic-induced early concentric hypertrophy remodeling: participation of a novel crosstalk between cardiac Mφ and cardiomyocytes. Early β-adrenergic stimulation activates the cardiac CX3CL1/CX3CR1 axis that supports early transient concentric remodeling and delays evolution towards heart failure: this is associated with a CX3CL1/CX3CR1-dependent expansion of cardiac Mφ. CX3CL1 and TNF-α secreted by cardiac Mφ synergistically trigger cardiomyocyte hypertrophy.

Expanding knowledge of the immune system's role provides opportunities for improving heart disease management. Our results demonstrate that the β-adrenergic-induced ECH heart exhibits a selective transient increase in Mφ as compared to control and HF hearts, with a Mφ number correlating with ECH intensity. Based on systematic echocardiographic analyses, our study provides a detailed characterization of iso-induced ECH myeloid cells using a combination of flow cytometry, tissue immunostaining, transcriptomic and secretomic approaches. Our in-vivo and in-vitro results highlight that Mφ-secreted CX3CL1 is a pro-proliferative and pro-hypertrophic factor and that the cardiac CX3CL1/CX3CR1 axis exerts a protective transient driving effect at the onset of ECH. Mechanistically, we show the synergistic action of CX3CL1 and TNFα, both secreted by ECH Mφ, and the potential role of the CX3CR1-dependent Mφ/cardiomyocyte crosstalk in ECH (Fig. 7).

Our results demonstrate that clodronate injection, at the onset of ECH, depletes Mφ, hampers ECH development and elicits direct progression towards cardiac dysfunction. The trancriptomic analysis with IPA profiles a biological signature of ECH Mφ consistent with a protective impact against HF, including down-regulation of inflammation, activation of phagocytosis, inhibition of fibrosis and up-regulation of leukocyte survival. Furthermore, IPA designates IFNγ as the top upregulated signaling pathway in the iso-induced ECH model. As a protective impact of the IFNγ axis has previously been reported in TAC-induced cardiac compensatory hypertrophy^26^, our study provides new evidence for a beneficial role of iso-induced Mφ in the early concentric hypertrophy response. In addition, we identify the predominant upregulated ECH Mφ subpopulation as Ly6c^low^/CCR2^-^. Ly6c^-^/CCR2^-^ Mφ are reported to protect against cardiovascular diseases in contrast with Ly6c^+^/CCR2^+^ Mφ that are generally considered as pro-inflammatory and deleterious^27^.

We identify CX3CL1 as one of the top upregulated genes in ECH Mφ as compared to control and HF counterparts. Our in-vitro results demonstrate the direct impact of CX3CL1 on the cardiac Mφ number and their proliferative activity. Of note, a proliferation of cardiac Mφ is detected in-vivo in ECH hearts. In contrast, our preliminary RNAseq analyses comparing Ct, ECH and HF blood leukocytes do not evidence any increase in *Ccr2* or *Cx3cr1* mRNA in response to early iso-infusion, arguing for a limited participation of recruited blood monocytes to the increased number of cardiac Mφ during ECH (not shown). In line, additional results from our laboratory indicate that WT mice and *Ccr2*^*-/-*^ counterparts subjected to iso-treatment for 14 days display similar PWs [108 ± 4 vs 101 ± 4% of control value at day0, in WT vs *Ccr2*^*-/-*^ mice, respectively], LVs [108 ± 8 vs 109 ± 6% of control value at day0, in WT vs *Ccr2*^*-/-*^ mice, respectively] and FS [93 ± 6 vs 89 ± 5% of control value at day0, in WT vs *Ccr2*^*-/-*^ mice, respectively] echocardiographic parameters. Thus, in contrast to *Cx3cr1*^*-/-*^ mice, *Ccr2*^*-/-*^ mice do not develop cardiac early dilation and dysfunction. Our in-vivo experiments show that knockout of CX3CR1, the unique CX3CL1 receptor, blunts the upregulation of Mφ number in response to a 14 days iso-infusion. This is associated with a lack of early concentric hypertrophy and a direct evolution towards dilation, alteration of the cardiac function, and increased apoptotic and fibrotic remodeling. SiRNA-mediated intramyocardial neutralization of either CX3CL1 or CX3CR1 also hampers the ECH response and accelerates cardiac dysfunction. Our study provides the first in-vivo data demonstrating the direct protective role of the CX3CL1/CX3CR1 axis to drive ECH.

The role of the CX3CL1 pathway in heart disease has been highly controversial due to conflicting reports on its circulating ligands and limited information regarding its functional consequences in the heart in-vivo. CX3CR1 knockout has been shown to aggravate Coxsackievirus B3-induced myocarditis^28^. Our results illustrate that inhibition of the CX3CL1/CX3CR1 axis hampers ECH remodeling and triggers HF. They argue for the participation of cardiac Mφ in this protective function. However, other studies have reported that CX3CL1 also increases endothelial and smooth muscle cell migration and proliferation and acts as a proangiogenic factor that favors neovascularization^21^. Of note, our preliminary results indicate the absence of modification of CD31^+^ staining in ECH hearts as compared to control hearts, suggesting a limited angiogenic remodeling in response to early iso-infusion. The membrane-bound CX3CL1 expressed by endothelial cells also serves as an adhesion molecule for the recruitment of leucocytes^29^ and favors cardiac allograft rejection^30^. In addition, the CX3CL1/CX3CR1 axis is a central player in inflammatory disorders such as atherosclerosis development^31^. The circulating level of CX3CL1 were correlated with New York Heart Association (NYHA) grades of HF also suggesting that the CX3CL1/CX3CR1 axis could contribute to HF pathology^32–34^. In this context, we propose that the CX3CL1/CX3CR1 pathway acts like a double-edge sword: it could exert a transitory compensatory impact early but become deleterious in HF. Such a dual effect is well documented concerning the neurohormonal systems.

Mechanistically, we demonstrate that, in contrast to in-vitro experiments performed in neonatal cardiomyocytes^34^, CX3CL1 alone is not sufficient to induce adult cardiomyocyte hypertrophy. We show that CX3CL1 acts in synergy with TNFα to trigger the adult cardiomyocyte concentric hypertrophy. Our results demonstrate that ECH adherent CD45^+^cells_,_ but not control or HF cells, are characterized by a secretion of both TNFα and CX3CL1. Several studies report a close interaction between TNFα and CX3CL1 pathways as, for instance, TNF-α has been shown to positively regulate the expression of CX3CL1^35–37^ or CX3CR1^20^. Moreover, CX3CL1 has also been reported to modulate TNFα expression^20, 38^, potentially limiting or enhancing TNFα secretion depending on the dose^39^. Therefore, our study further documents a novel early beneficial role of TNFα in ECH, relying on the binding to TNFR_1_, and a combined action with CX3CL1 to initiate hypertrophy in control cardiomyocytes. We previously identified the Orai3-dependent TNFα pathway, selectively active in hypertrophied cardiomyocytes but not in control cells, as promoting hypertrophy and increasing resistance to oxidative stress through TNFR_2_^15^. Interestingly, the present newly identified TNFα-TNFR1/CX3CL1-dependent pro-hypertrophic mechanism is unaffected by pretreatment with an Orai3-inhibitor suggesting a novel and Orai3-independent pathway (not shown). Therefore, the present and previous studies^15^, further support that TNFα also behaves as a transitory beneficial compensatory pathway in ECH that is well known to become maladaptive in HF.

Clinical studies have clearly established that any abnormal change in LV geometry (concentric or eccentric) is associated with increased risk of cardiovascular diseases^40^. It is well established that β‐adrenoceptor signaling plays a central role in cardiac hypertrophy and maladaptation, in particular following pressure overload^41^. Concentric hypertrophy is described as an early adaptive response to an original cardiac stress to maintain a normal systolic function. However, ageing, gender, increased blood pressure and BMI are key clinical risk factors of dynamic worsening. Data in humans regarding the development of LV geometric pattern over time are relatively scarce. However, there is substantial evidence for a potential temporal sequence of transient concentric hypertrophy evolving at long term towards eccentric hypertrophy and the development of HF^42,43^, as already suggested in animal studies and supported by the present results.

In conclusion, our results highlight a crosstalk between Mφ and cardiomyocytes to induce ECH remodeling and the causal role of the CX3CL1/CX3CR1 axis in this intercellular communication. Therefore, in the context of recent studies supporting the crucial contribution of nonmyocytes to the early cardiac adaptive response, our data strengthen the concept that cardiomyocytes and Mφ may form a network and dialogue with each other to shape the cardiac tissue^44^. This work opens new avenues for medication of cardiac diseases.

## Supplementary Information

Supplementary Information.

## Abbreviations

ECH: Early concentric hypertrophy
HF: Heart failure
Mφ: Macrophages
Iso: Isoproterenol
Cmed: Conditioned medium
Cardio: Cardiomyocytes
