# Supplementary material for: Early activation of the cardiac CX3CL1/CX3CR1 axis delays β-adrenergic-induced heart failure
Source: Sci Rep. 2021 Sep 9;11:17982. doi: 10.1038/s41598-021-97493-z (PMC8429682; doi:10.1038/s41598-021-97493-z)
Supplement: Supplementary file 1 — Supplementary Information. [file 41598_2021_97493_MOESM1_ESM.pdf]

Early activation of the cardiac CX3CL1/CX3CR1 axis delays β-adrenergic-induced heart failure

Flamant M, PhD[1], Mougenot N, PhD[2], Balse, E, PhD[1], Le Fèvre L, MSC[1], Atassi F, BSC[1], Gautier EL, PhD[3], Le Goff W, PhD[4], Keck M, PhD[1], Nadaud S, PhD[1], Combadière C, PhD[5], Boissonnas A, PhD[5] and Pavoine C, PhD[1*].

[1]Sorbonne Université, UPMC Univ Paris 06, INSERM, Institute of Cardiometabolism and Nutrition (ICAN), Team 3, F-75013, Paris, France.

[2]Sorbonne Université, UMS28, Plateforme d'Expérimentation Cœur, Muscles, Vaisseaux (PECMV), F-75013, Paris, France.

[3]Sorbonne Université, UPMC Univ Paris 06, INSERM, Institute of Cardiometabolism and Nutrition (ICAN), Team 5, F-75013, Paris, France.

[4]Sorbonne Université, UPMC Univ Paris 06, INSERM, Institute of Cardiometabolism and Nutrition (ICAN), Team 4, F-75013, Paris, France.

[5]Sorbonne Université, Inserm, CNRS, Centre d'Immunologie et des Maladies Infectieuses CIMI-Paris, F-75013, Paris, France.

Short Title: protective CX3CL1/CX3CR1 axis in heart failure

Address for corresponding author*: Catherine Pavoine, PhD
UMR_S ICAN 1166 Team 3
91 bd de l'hôpital
75013 Paris
France
Phone : +33 1 40 77 96 62
Email : catherine.pavoine@inserm.fr

Current addresses of authors who have moved:

Le Fèvre L. Medical and infectious intensive care unit, Bichat hospital, APHP, 46 rue Henri Huchard 75018 Paris

Keck M. Université Paris-Saclay, CEA, INRAE, Département Médicaments et Technologies pour la Santé (DMTS), SIMoS, 91191 Gif-sur-Yvette, France

Methods

Ethics

Care of the animals and surgical procedures were performed according to the Directive 2010/63/EU of the European Parliament, which had been approved by the Ministry of Agriculture, France, (authorization for surgery C-75-665-R). The project was submitted to the French Ethic Committee CEEA (*Comité d'Ethique en Expérimentation Animale*) and obtained the authorization Ce5/2012/050 and APAFIS#1729-2015-083114195840v8. All experiments were performed in accordance with relevant named guidelines and regulations and in compliance with the ARRIVE guidelines.

Isoflurane was used to anesthetize mice during echocardiography analysis (0.2-0.5 %), intramyocardial siRNA injections (1 %), and Alzet micropump implantation (2-3 %). The adequacy of anesthesia was confirmed by the absence of a reflex response to foot squeeze. Mice were euthanized via cervical dislocation and hearts were isolated for further histological and protein analyses or before cardiomyocyte or macrophage isolations.

Animals

Experiments were conducted on adult male mice (9-20 week-old) of the following strains: C57BL/6J mice purchased from Janvier Labs (Le Genest St Isle, France); *Cx3cr1*$^{-/-}$ mice (C57BL/6J genetic background, as previously reported [1]) bred at Pitié-Salpétrière animal facility.

In-vivo chronic isoproterenol infusion

Mice were implanted with an osmotic micropump (Alzet, Charles River, L'Arbresles, France) containing either isoproterenol (iso) (30 mg/kg/day) or vehicle for either 14 or 28 days to develop either ECH or HF, respectively, as previously reported [2], and as attested by systematic echocardiographic analyses. Of note, there was a slight but possible variability in the kinetics or in the maximum intensity of cardiac remodeling triggered by iso: for this reason, protocols always included a group of control animals (WT or untreated or control injected) in parallel, to allow comparison. More frequently, we observed a reduced or delayed cardiac remodeling and a potential absence of HF at day 25-28 iso in experiments involving repeated injections of either liposomes or PBS: our hypothesis was that repeated injections could generate a low-grade inflammatory response capable of limiting or delaying deleterious cardiac remodeling.

In-vivo intramyocardial ultrasound-guided transthoracic siRNA delivery in mice

SMART pool On-target plus Scramble or *Cx3cl1* or *Cx3cr1* siRNA (Dharmacon, Cambridge, UK) were injected by ultrasound-guided transthoracic intramyocardial injection (see Figure S9) in mice (solution 2.5µM in lipofectamine/optimem, according to the manufacturer instructions, 80µl/mice), as described in Keck et al.[2], at day 7 after iso pump implantation. Echocardiographic parameters were measured regularly as stated.

Measurement of cardiac parameters

Echocardiography was performed on lightly anesthetized animals under isoflurane (0.2-0.5 %) with a probe emitting ultrasounds from 9- to 14-MHz frequency (Vivid7 PRO apparatus; GE Medical System Co), as previously reported[2]. The two-dimensionally guided Time Motion mode recording (parasternal long-axis view) of the left ventricle (LV) provided the following measurements: end-diastolic and end-systolic interventricular septum (IVSd and IVSs), posterior wall thicknesses (PWd and PWs), internal diameter (LVEDD and LVESD), and heart rate (HR). Each set of measurements was obtained from the same cardiac cycle. At least three sets of measurements were registered from three different cardiac cycles. Fractional shortening (FS): [(LVEDD − LVESD)/LVEDD] × 100 and h/r: [left ventricle diastolic wall thickness / radius] were calculated. A conserved FS, with a thickening of PWs in the absence of an increase in LVEDs characterized ECH.

Cardiomyocyte isolation and culture

Cardiomyocytes were isolated from adult mice using a simplified Langendorff-free method, introducing *ex vivo* optimized dissociation buffers to the heart by direct needle injection into the left ventricle, as previously reported [2]. After cutting of the descending aorta and inferior vena cava, the heart is immediately flushed with cold EDTA buffer (130 mM NaCl, 5 mM KCl, 0.5 mM NaH2PO4, 10 mM HEPES, 10 mM glucose, 10 mM 2,3-butanedione monoxime (BDM), 10 mM taurine, 5 mM EDTA pH 7.8), by injection in the right ventricle. After clamping of the emerging aorta, the heart is transferred to 60mm dishes for subsequent injection and digestion steps. Deep myocardial perfusion via the coronary vasculature is induced by injection in the left ventricle, first with EDTA buffer for 6 min, then with a Perfusion buffer for 2 min (130 mM NaCl, 5 mM KCl, 0.5 mM NaH2PO4, 10 mM HEPES, 10 mM glucose, 10 mM BDM, 10 mM taurine, 1 mM MgCl2 pH 7.8). Digestion is performed in a collagenase buffer for 30 min at 37°C using an increasing flow rate from 0.5 to 2.5 ml/min (2.5

mg/mL collagenase 2 (Worthington) dissolved in perfusion buffer). A Stop buffer is made with perfusion buffer containing 5% sterile fetal bovine serum (FBS) to stop collagenase action. Isolated cardiomyocytes are then purified by sequential gravity settling steps, with a gradual calcium reintroduction to produce calcium-tolerant cells. The protocol reproducibly yields 655000 ±156000 total cells/heart and 46 ± 3% viable, rod-shaped cells.

Cardiomyocytes were plated onto laminin-coated wells (5 µg/ml, Roche) at a density of 30000 total cells/ml in a plating medium (M199 medium (Life Technologies, Courtaboeuf, France) / Joklik medium (1/1 vol/vol) added with 10mM BDM, 1% penicillin-streptomycin (PS), 1% insulin/transferrin/selenium (ITS) and 5% FBS). They were let to adhere for 3 h before treatments with either CX3CL1 or TNFα or both, conditioned medium (Cmed) from Mφ-enriched adherent $CD45^+$cells ± pharmacological inhibitors (with RPMI medium as internal control, prepared as described below), or ± CX3CL1 or $TNFR_1$ or $TNFR_2$ antibodies (Abs, when stated, in a culture medium (idem plating medium but with only 1.5% FBS) and maintained overnight. Each experimental condition was evaluated in triplicate.

Measurement of cardiomyocyte hypertrophy

Cardiomyocytes were visualized using brightfield at x20 magnification and cell width, length and area were measured in at least 300 cells per condition per experiment. Results were the mean of at least three different experiments performed on two cell isolations (using at least 4 different Cmed from adherent $CD45^+$cells).

Isolation of cardiac immune cells for Conditioned media (Cmed) preparation and in-vitro proliferation assay

After perfusion with PBS, the mice heart was excised and digested in HBSS medium containing 2.5 mg/ml collagenase D (Roche, Meylan, France) for 30 min at 37°C, with stirring. Erythrocytes were lysed by using red blood cell lysis buffer (MiltenyiBiotec, Paris, France). Cardiac CD45 cells were isolated by centrifugation, enriched using an anti-CD45 antibody coupled to magnetic beads (MiltenyiBiotec, Paris, France) and all $CD45^+$ cells isolated from each heart were systematically seeded in 2 wells from a 48 multiwell plate (final volume 500µl in a RPMI medium (Life Technologies, Courtaboeuf, France) supplemented with 10 mmol/LHepes). After 3h of adhesion, the medium was renewed (this allowed elimination of non-adherent cells such as lymphocytes or NKT cells) and adherent cells (containing an average of 64.2 ± 3.5 % CD64 positive cells) were incubated overnight before collection of their conditioned medium (Cmed). The Cmed was concentrated 3 times on Amicon 3kDa Ultra

centrifugal filter (Millipore), kept at -80°C until *in-vitro* studies on cardiomyocyte hypertrophy and used at a 1/20 final dilution. RPMI medium treated in parallel and the same way as Cmed was used as internal experimental control. Cytokines and chemokines in the Cmed were quantified using a bio-plex immunoassay (Biorad, Marnes-La-Coquette, France). For *in-vitro* proliferation assays, Mφ were kept in culture for 48 hours in the presence or absence of CX3CL1 (50ng/ml) before fixation in paraformaldehyde and fluorescent staining.

Quantification of macrophage proliferation

For BrdU incorporation assays, mice were injected intraperitoneally with 1 mg BrdU (Santa Cruz, Heidelberg, Germany) in 100µl PBS 2 hours prior to sacrifice and organ harvest. Intracellular staining was performed using the anti-BrdU antibody (Abcam, Paris, France, 1/100 dilution) after acid treatment to unwind the DNA and help antibodies access to DNA incorporated BrdU. After neutralization with sodium borate, anti-BrdU Ab was revealed with goat anti-rat Alexa fluor 488 (Abcam, Paris, France, 1/500 dilution). Macrophages were then stained with an anti-CD68 Ab (Biolegend, Saint-Quentin-En-Yveline, France, 1/200 dilution) revealed with goat anti-rat Alexa fluor 555 (Abcam, Paris, France, 1/500 dilution). DAPI and WGA stained nuclei and membranes, respectively.

Isolation and Preparation of immune cells for flow cytometry analysis

After perfusion with PBS, the mice heart was excised and digested in HBSS medium containing 2.5 mg/ml collagenase D (Roche, Meylan, France) for 30 min at 37°C, with stirring.

Erythrocytes were lysed by using red blood cell lysis buffer (MiltenyiBiotec, Paris, France). Samples were blocked with Fc block (Ebioscience, Paris, France) prior to labeling with antibodies. Cytometry data were acquired on an LSR Fortessa cytometer. After gating on $CD45^+$ cells, doublets were excluded and live cells were analyzed (PI exclusion). Cardiac cell numbers were quantified using polybeads (Polysciences, Le-Perray-en-Yvelines, France). Data were analyzed with FlowJo software (Tree Star).

Preparation of immune cells for fluorescence activated cell sorting

Cardiac immune cells were isolated by centrifugation, enriched by immunoselection using an anti-CD45 antibody coupled to magnetic beads (MiltenyiBiotec, Paris, France). Samples were blocked with Fc block (Ebioscience, Paris, France) prior to labeling with antibodies. Cytometry data were acquired on a BD FACSAria II cell sorter (5 lasers). After gating on CD11b+ cells, doublets were excluded and live (PI exclusion) $CD14^+$/$CD64^+$ Mφ

were sorted directly into RLT lysis buffer (Qiagen) or water and kept at -80°C until RNAseq analysis.

Antibodies / flow cytometry / sorting / western blotting

experiment	Target	Clone	Isotype	Reference	Dilution	Fluorochrome	Source
analysis	CD11b	REA-592	Human IgG1	130-109-290	1 : 80	Vio Bright FITC	MiltenyiBiotec Paris, France
Analysis	CD45	30-F11	Rat IgG2b, κ	48-0451-82	1 : 200	eFluor 450	Ebioscience Paris, France
analysis & sorting	CD64	REA-286	Humain IgG1	130-103-808	1 : 40	PE	MiltenyiBiotec Paris, France
analysis & sorting	MHC-II	M5/114.15.2	Rat IgG2b, κ	56-5321-82	1 : 400	Alexa Fluor 700	Ebioscience Paris, France
analysis	CCR2	475301	Rat IgG2b	FAB5538A	1 : 10	APC	R&D Systems Abingdon UK
analysis	Ly6C	AL-21	Rat IgM, κ	560596	1 : 400	APC-Cy 7	BDBiosciences Le-Pont-De-Claix, France
sorting	CD11b	M 1/70	Rat IgG2b, κ	48-0112-82	1 : 200	eFluor 450	Ebiosciences Paris, France
sorting	CD14	SA2-8	Rat IgG2a, κ	11-0141-82	1 : 200	FITC	Ebiosciences Paris, France
WB neutralization	CX3CL1	polyclonal	Rabbit IgG	TP233	1 : 1000		Torrey Pines Secaucus USA
WB	CX3CR1	polyclonal	Rabbit IgG	Ab8021	1 : 1000		Abcam, Paris, France
WB	TNFα	EPR20972	Rabbit	Ab215188	1 : 1000		Abcam, Paris, France
WB	GAPDH	14C10	Rabbit	2118	1 :25000		Cell signal, St-Cyr, France

RNA sequencing and statistical analysis:

Total RNA from FACS sorted $CD64^+/CD14^+$ cells was isolated using the Nucleospin RNA XS kit (Macherey Nagel, Hoerdt, France), according to the manufacturer instructions. cDNA libraries were generated using total RNA with SMART-Seq v4 Ultra Low Input RNA Kit (TAKARA) and constructed according to manufacturer protocols as previously reported [3]. Paired end sequencing (2 x 750 bp) was performed by Nextseq 500 machine using High Output kit (150 cycles). Raw sequencing data was quality-controlled with the FastQC program. Trimmomatic was used to remove adapter sequences, trim low quality reads, and discard reads

shorter than 40 bp. Reads were aligned to the mouse reference genome (build mm10) with the TopHat2 tool. Mapping results were quality-checked using RNA-SeQC. Aligned reads were counted using the FeatureCounts and Express software, at the gene-level and transcript-level, respectively. Normalization and differential analysis were performed with the GLM EdgeR package. RNA-Seq data has been made publicly available through the NCBI Gene Expression Omnibus (GEO), GEO accession number GSE157035.

Quantitative RT-PCR

Total RNA from CD64$^+$ sorted cells was isolated with the Nucleospin RNA XS kit (Macherey Nagel). RNA reverse transcriptase-PCR analysis was performed using Verso cDNA Synthesis kit (Thermo Scientific) on a Lightcycler 1536 DNA green master (Roche) and a Bravo system (Agilent Technologies, Massy, France).

Primer sequences are listed below. Transcript levels were normalized to the *Rpl13* mRNA.

**Gene**		Sequence (5'→3')
Cx3cl1	C-X3-C motif chemokine ligand 1	CGCGTTCTTCCATTTGTGTA
		CTGTGTCGTCTCCAGGACAA
Rpl13	Ribosomal protein L13	GAGGAGGCGAAACAAGTCCA
		GGGTGGCCAGCTTAAGTTCT

Immuno-fluorescence

Immuno-fluorescence studies were performed on frozen sections or cells fixed in paraformaldehyde as described in[2]. Immune cells or Frozen cardiac sections (6µm) fixed in paraformaldehyde for 15 min at room temperature and were stained immuno-histochemically with the following antibodies: rabbit anti-mouse Ki67 antibodies revealed with goat anti-rabbit Alexa fluor 488 (1/500, Thermo Scientific, Montigny-Le-Bretonneux, France, anti-CD68 (rat, 1/200, Biolegend, Paris, France) revealed using Alexa fluor 488 (goat anti-rat, 1/500, Abcam, Paris, France) or Alexa fluor 555 (goat anti-rat, 1/500, Abcam, Paris, France) or Alexa fluor 647 (goat anti-rat, 1/500, Abcam, Paris, France) or Alexa fluor 488 (donkey anti-rat, 1/500, Abcam, Paris, France), anti-CD64 (rat, 1/500, Interchim, Montluçon, France) revealed using Alexa fluor 488 (donkey anti-rat, 1/500, Abcam, Paris, France), anti-Mer (goat, 1/200, R&D systems, Lille, France) revealed using Alexa fluor 555 (donkey anti-goat, 1/500, Abcam, Paris, France), anti-cleaved caspase 3 (rabbit polyclonal, 1/50, R&D systems, Abingdon, UK) revealed using Alexa fluor 546 goat anti-rabbit (1/500, Thermo-Scientific, Montigny-Le-

Bretonneux, France); anti-CX3CL1 (rabbit polyclonal, 1/100, Torrey Pines, Clinisciences, Nanterre, France) revealed using Alexa fluor 546 (goat anti-rabbit, 1/500, Thermofisher, Montigny-Le-Bretonneux, France); anti-CX3CR1 (rabbit polyclonal, 15µg/ml, Abcam, Paris, France) revealed using Alexa fluor 647 (donkey anti-rabbit, 1/500, Abcam, Paris, France); after saturation of mouse cardiac tissues with anti-mouse IgG (H+L) (donkey, 100µg/ml, Jackson Immuno research, Newmarket, UK), anti-TNFα (mouse polyclonal, 1/150, Abcam, Paris, France) revealed using Alexa fluor 488 (goat anti-mouse, Invitrogen, Paris, France). Membranes were labeled with wheat germ agglutinin (WGA)-Alexa 647 (1/500, Thermo Scientific, Montigny-Le-Bretonneux, France) and nuclei with DAPI.

Tissue sections and cells were analyzed with a Zeiss Axio Observer Z1 microscope. Image analysis was performed using ImageJ and Photoshop CS5 (Adobe, San Jose, CA, USA). Results are expressed as the number of positive cells per field and were quantified from 4-10 mice per group and 20-32 images per animal.

Quantification of cardiomyocyte area and tissue fibrosis

Frozen sections fixed in paraformaldehyde were labeled with WGA-Alexa 647 (1/500 dilution, Thermo Scientific, Montigny-Le-Bretonneux, France). Tissue sections were analyzed with a Zeiss Axio Observer Z1 microscope using ImageJ software. A low *vs.* high threshold allowed quantification of cardiomyocyte area or tissue fibrosis, respectively, as previously reported [2]. Results were quantified from 6-7 mice/group (12-32 images/animal).

Western Blot

Isolated cardiomyocytes or tissue homogenates were lysed in 150 mM NaCl, 50 mM Tris pH=7.4, EDTA 1 mM, EGTA 1 mM, Na4P2O7 5 mM, 10% glycerol, 1% triton, 10% glycerol and protease and phosphatase inhibitors cocktail (Sigma, St-Quentin-Fallavier, France). Samples were then centrifuged at 3000 x *g* for 5 minutes to get rid of cell debris. Protein concentration was measured by the BCA Protein assay (Thermo Scientific, Montigny-Le-Bretonneux , France).

Proteins were separated on NuPAGE Novex 10% or 4-12% Bis-Tris gels (Life Technologies) and transferred on nitrocellulose membrane (Biorad, Marnes-La-Coquette, France). Membranes were cut and each part was incubated with indicated antibodies. Incubation

were performed with appropriate primary antibodies (see antibodies listing above) followed by HRP-coupled secondary antibodies (Cell Signal, St-Cyr, France). Proteins were revealed with ECL Prime (GE Healthcare, Velizy, France) and images were acquired using a LAS4000 Camera (GE Healthcare, Velizy, France).

We displayed cropped gels and blots in the main paper to improve the clarity and conciseness of the presentation. However, full-length unedited material was provided in the supplementary information, as mentioned in the figure legends.

Drugs

CX3CL1 and TNFα (R&D Systems, Lille, France) were used at 50 or 100 ng/mL. Neutralizing anti-$TNFR_1$ Abs (Hycult Biotech, Clinisciences, Nanterre, France), anti-$TNFR_2$ Abs (Biolegend, Paris, France) or anti-CX3CL1 Abs (Torrey Pines Biolabs, Secaucus, USA) were used at a final concentration of 1 µg/mL, 10 µg/mL or 20 µg/mL, respectively and preincubated for 30 min at 37°C. Control or clodronate liposomes (VU University Medical Center, Amsterdam, The Netherland) were used *in-vivo* at 100 µl/25 g by i.p. injections three times a week beginning at day 7 following pump implantation and until sacrifice at day 25. M-CSF (Eurobio, Les Ulis, France) was used at 50 ng/mL.

Statistical analysis

Quantitative data are reported as means ± SEM. Statistical analysis was performed with GraphPad Prism 6 (GraphPad software Inc, San Diego, CA, USA). For multiple comparisons of values D'agostino-and-Pearson normality test was first performed, and Kruskal-Wallis test, one-way ANOVA or 2-way ANOVA were used as appropriate followed by a post-hoc test for pairwise multiple comparisons. Correlation studies were performed using the Spearman correlation coefficient. Survival analysis was performed using the Gehan-Breslow-Wilcoxon test. All values with $p<0.05$ were considered significant.

References

1. Combadière C, Potteaux S, Gao J-L, Esposito B, Casanova S, Lee EJ, Debré P, Tedgui A, Murphy PM, Mallat Z. Decreased atherosclerotic lesion formation in CX3CR1/apolipoprotein E double knockout mice. *Circulation*. 2003;107:1009–1016.

2. Keck M, Flamant M, Mougenot N, Favier S, Atassi F, Barbier C, Nadaud S, Lompré A-M, Hulot J-S, Pavoine C. Cardiac inflammatory CD11b/c cells exert a protective role in hypertrophied cardiomyocyte by promoting TNFR 2 - and Orai3- dependent signaling. *Scientific Reports*. 2019;9:6047.

3. Song Y, Milon B, Ott S, Zhao X, Sadzewicz L, Shetty A, Boger ET, Tallon LJ, Morell RJ, Mahurkar A, Hertzano R. A comparative analysis of library prep approaches for sequencing low input translatome samples. *BMC Genomics*. 2018;19:696.

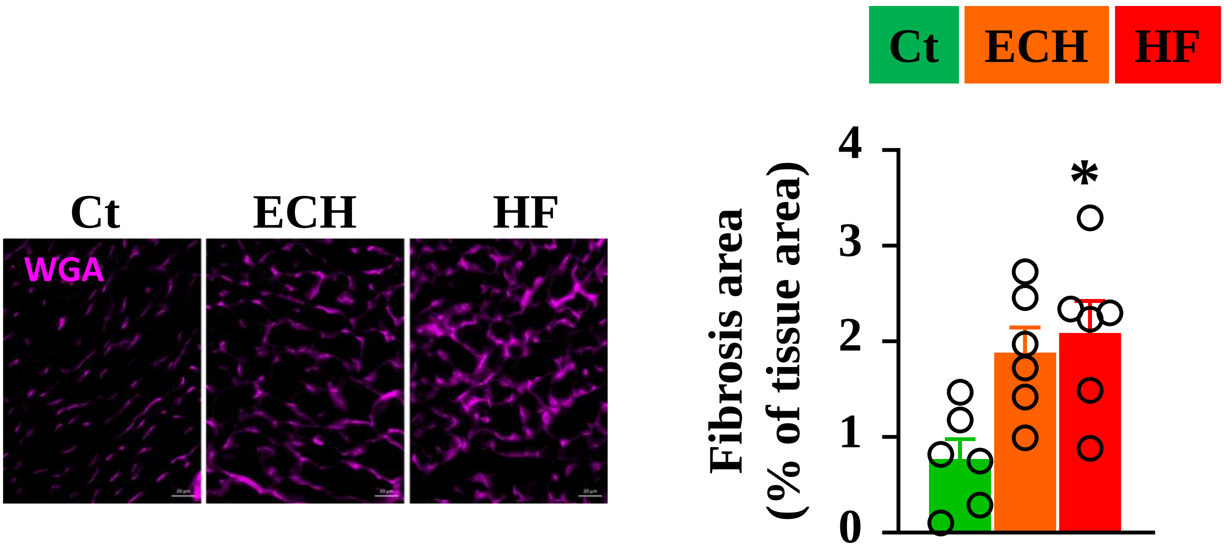

Figure S1: **HF hearts display higher fibrosis as compared to ct hearts**. Fibrosis area estimated in cardiac sections stained with WGA (typical images). Mean±SEM from 6 mice/group, sacrificed at day 0 (Ct), 14 (ECH) or 28 (HF) isoproterenol infusion, 32 images/mice, Kruskal-Wallis followed by Dunn's post-hoc test; * $p<0.05$. scale bar 20µm.

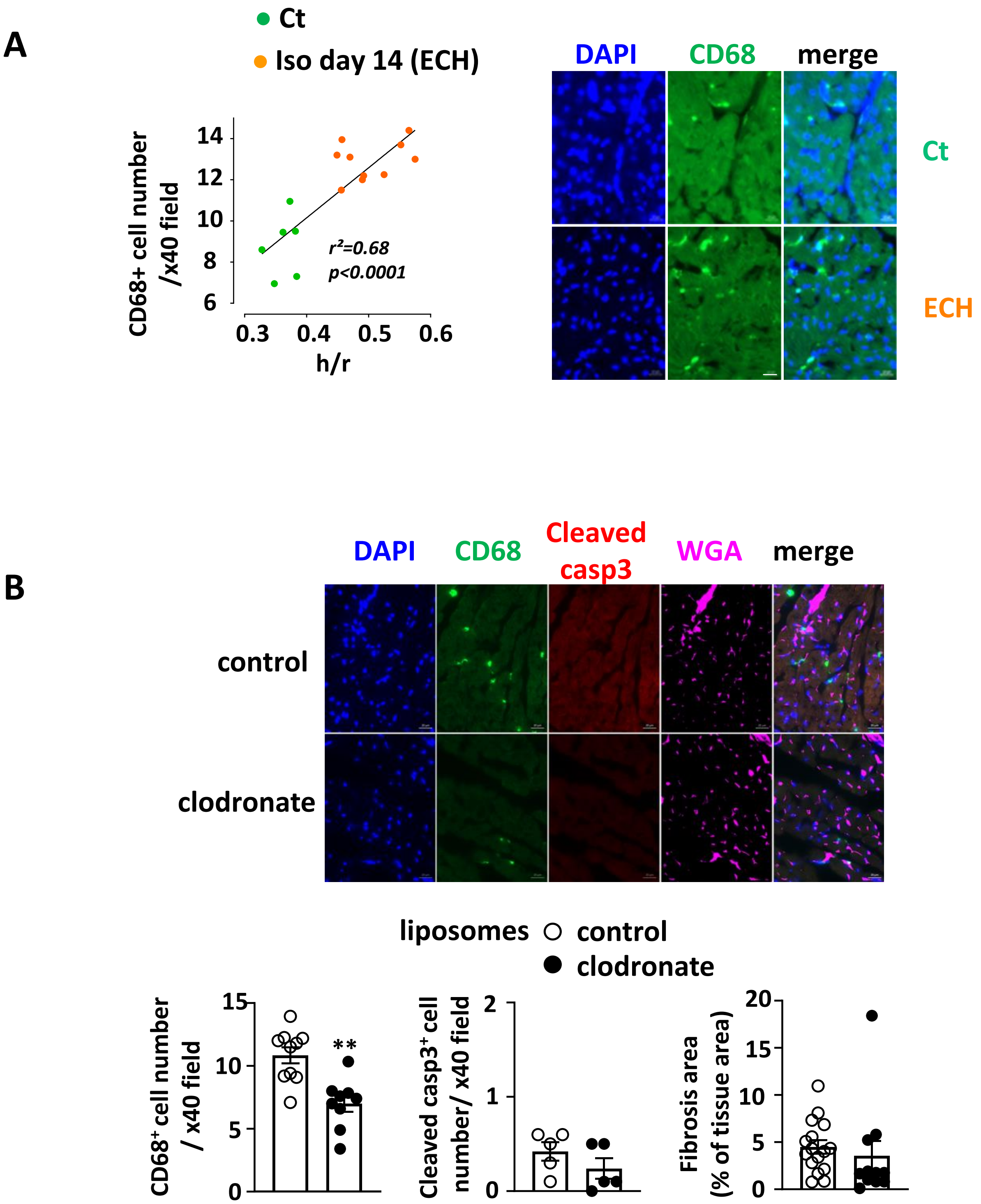

Figure S2: **An increase in CD68^{+} Mφ characterizes the early iso-infused hearts and correlates with concentric hypertrophy**. **(A)** Immuno-histofluorescence staining of Ct and ECH (Iso day14) cardiac tissues: correlation between cardiac CD68^{+} cell number and h/r; n=6 mice (Ct), n=10 mice (ECH), 20 images/mice. Scale bar 20µm. Correlation analysis using the Spearman correlation coefficient. **(B) Efficient depletion of cardiac CD68^{+} Mφ upon clodronate treatment. No impact of clodronate on either apoptosis or iso-induced fibrosis.** Typical images of cardiac sections from control or clodronate liposomes treated mice stained for CD68, Cleaved caspase3, WGA and Dapi and quantification of CD68^{+} and cleaved caspase3^{+} cell number or fibrosis area; n=9-10 mice/group, 20-27 images/mice, Mann-Whitney U test; * p<0.05. scale bar 20µm.

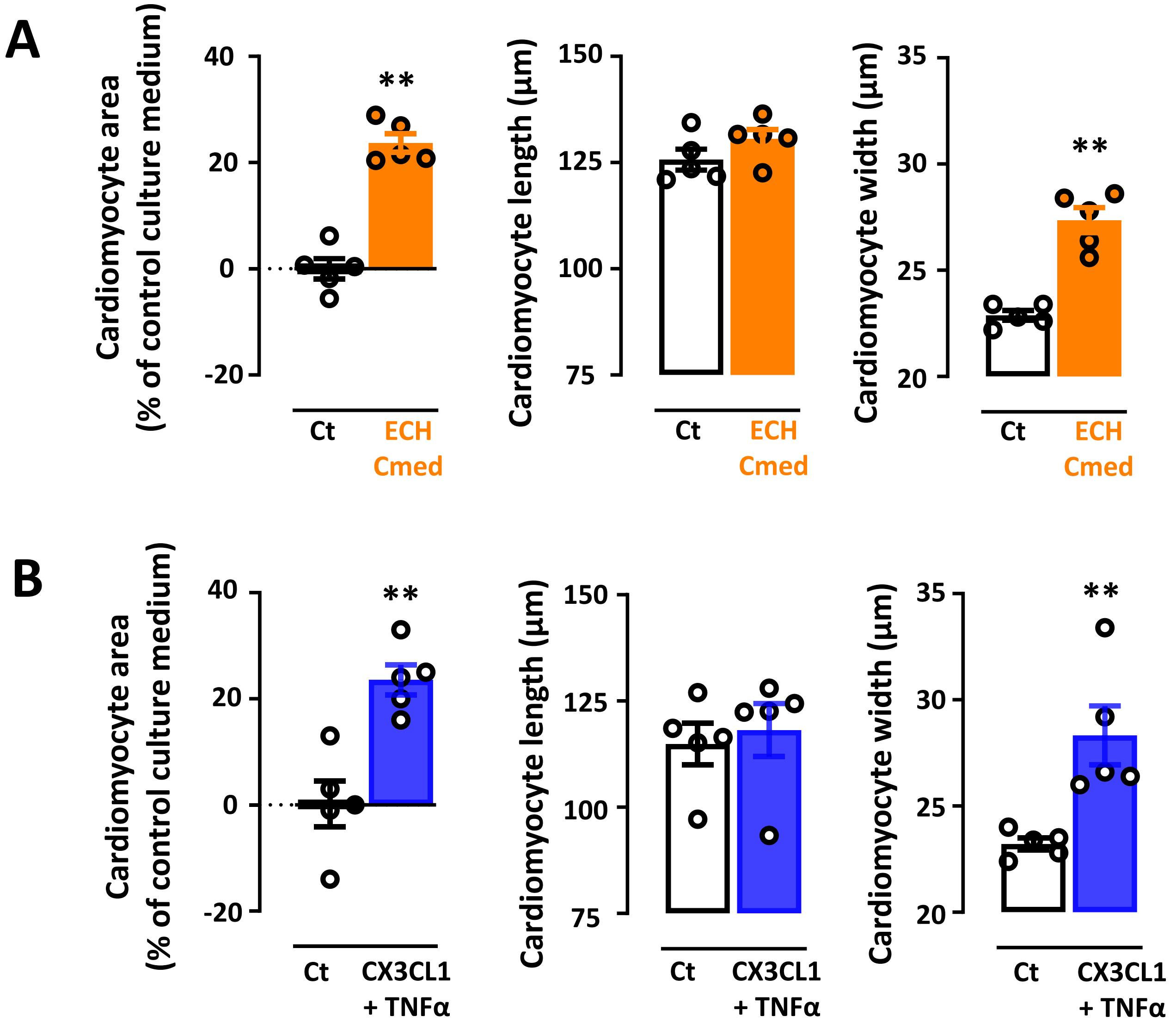

Figure S3: **Conditioned media from adherent CD45+ cells isolated from the iso-infused ECH hearts or the combination of CX3CL1 and TNFα enhance concentric hypertrophy in adult cardiomyocytes.**

Cardiac adherent (Mφ-enriched) CD45+ cells were isolated from iso-infused WT mice. Conditioned media (recovered after 18 h) or CX3CL1 + TNFα were applied on cardiomyocytes from WT mice. Cell area, length and width were analyzed 18 h later. **(A)** Cmed isolated from iso-infused ECH WT mice **(B)** CX3CL1 + TNFα. Mean±SEM of 3-5 experiments performed in triplicate. Cardiomyocytes from 3-5 mice (570-690 cell quantified per condition per experiment), Cmed from 5 mice, Mann Withney test. ** p<0.01.

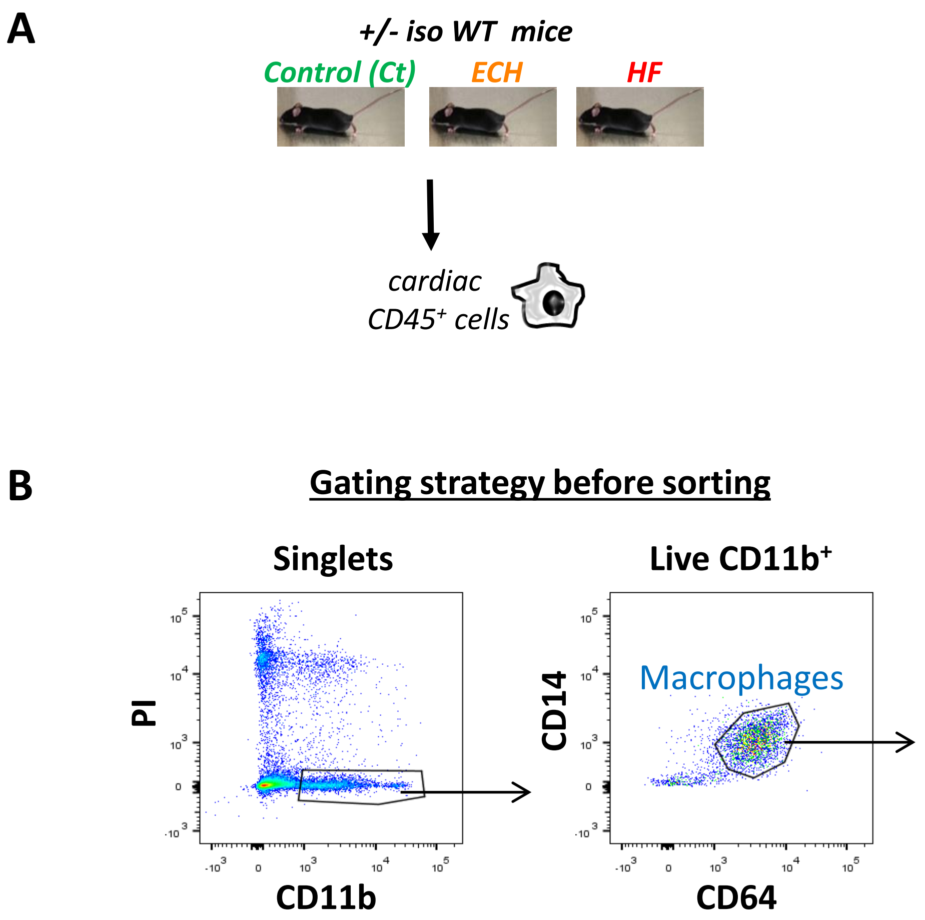

Figure S4: **Sorting of cardiac CD64^{+} Mφ before RNA sequencing.** Mice implanted or not with an iso-pump for 14 or 28 days were subjected to echocardiographic analyses. **(A)** Schematic representation of the protocol. **(B)** Typical flow cytometry gating strategy to sort cardiac CD14^{+}/CD64^{+} Mφ. Cardiac immune cells were isolated from collagenase digested hearts. Following CD45 positive enrichment using mouse CD45 microbeads, cells were stained with the indicated antibodies. Doublets were excluded (by FSC-W vs. SSCA) and live CD11^{+} cells (after PI exclusion) were gated on CD14^{+}/CD64^{+} Mφ and sorted.

A

IPA analysis: enriched in ECH macrophages

Canonical pathways	p-value
Th2 pathways	4.96E-04
Inhibition of MMPs	5.47E-04
Phagosome formation	1.11E-03

Functional analysis	Gene no	p-value	z-score
Quantity of connective tissue	24	8.26E-07	2,619
Chemotactism	12	6.45E-05	2,235
Phagocytosis	7	9.57E-03	0,677
Inflammation	31	3.50E-07	-3,039

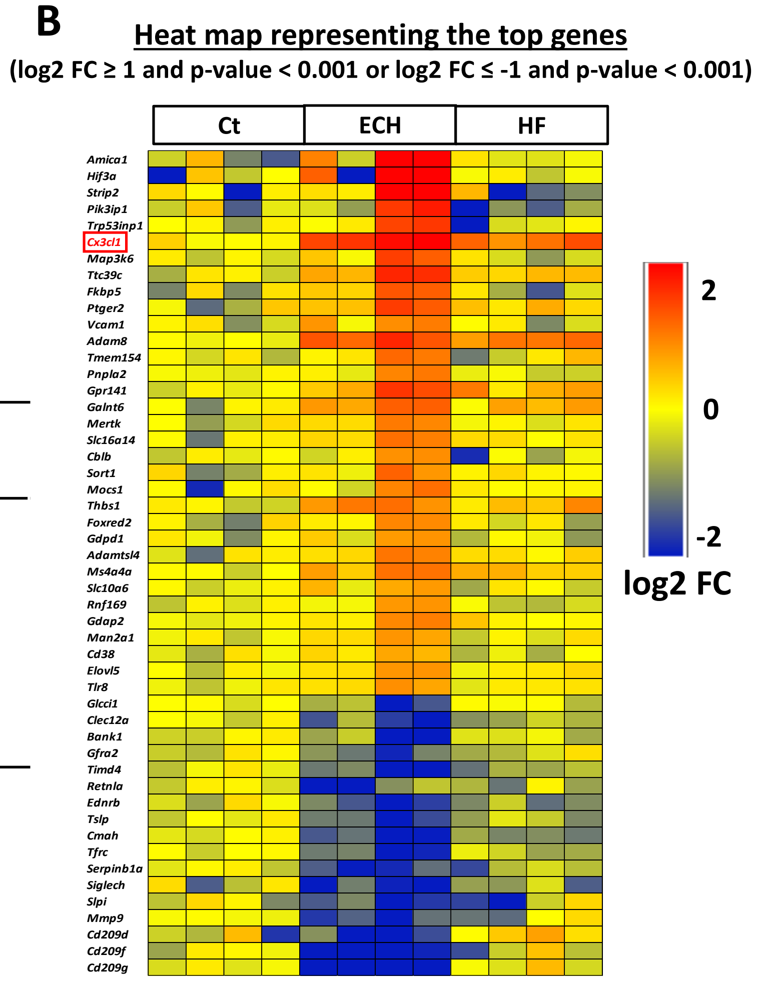

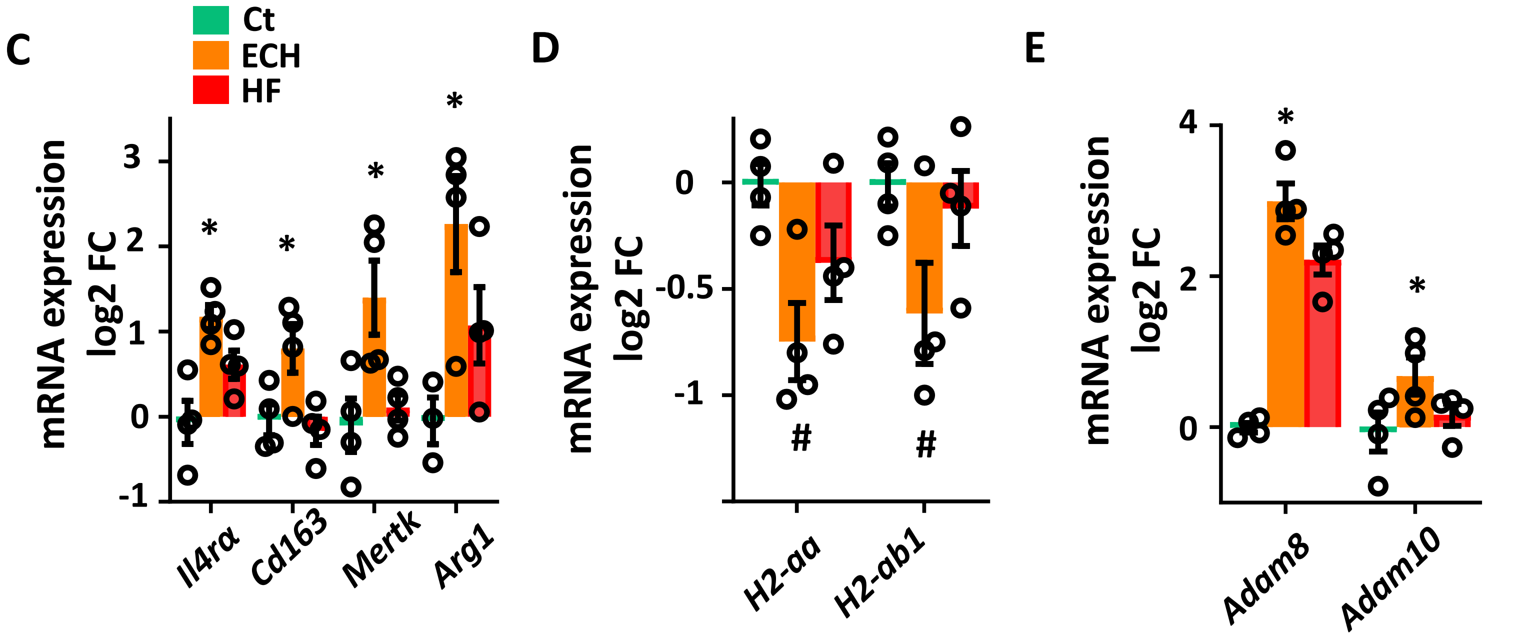

Figure S5: **Transcriptomic characterization of cardiac ECH CD64⁺ Mφ as compared to Ct and HF counterparts; (A)** Ingenuity Pathway analysis showing specific canonical pathways and functions selectively enriched in ECH Mφ. **(B)** Heat map showing the top genes selectively regulated in ECH Mφ as compared to Ct and HF counterparts (with red and blue indicating increased and decreased expression, respectively). n=4 mice/group. Normalization and differential analysis were performed with the glm edgeR package. RNAseq analysis. **(C)** phagocytosis- and M2-associated-gene expression. **(D)** *H2-aa* and *H2-ab* gene expression. **(E)** *Adam8* and *Adam10* gene expression; * p<0.05 ECH vs Ct and HF. # p<0.05 ECH vs Ct. N=4 mice/group, normalization and differential analysis were performed with the glm edgeR package.

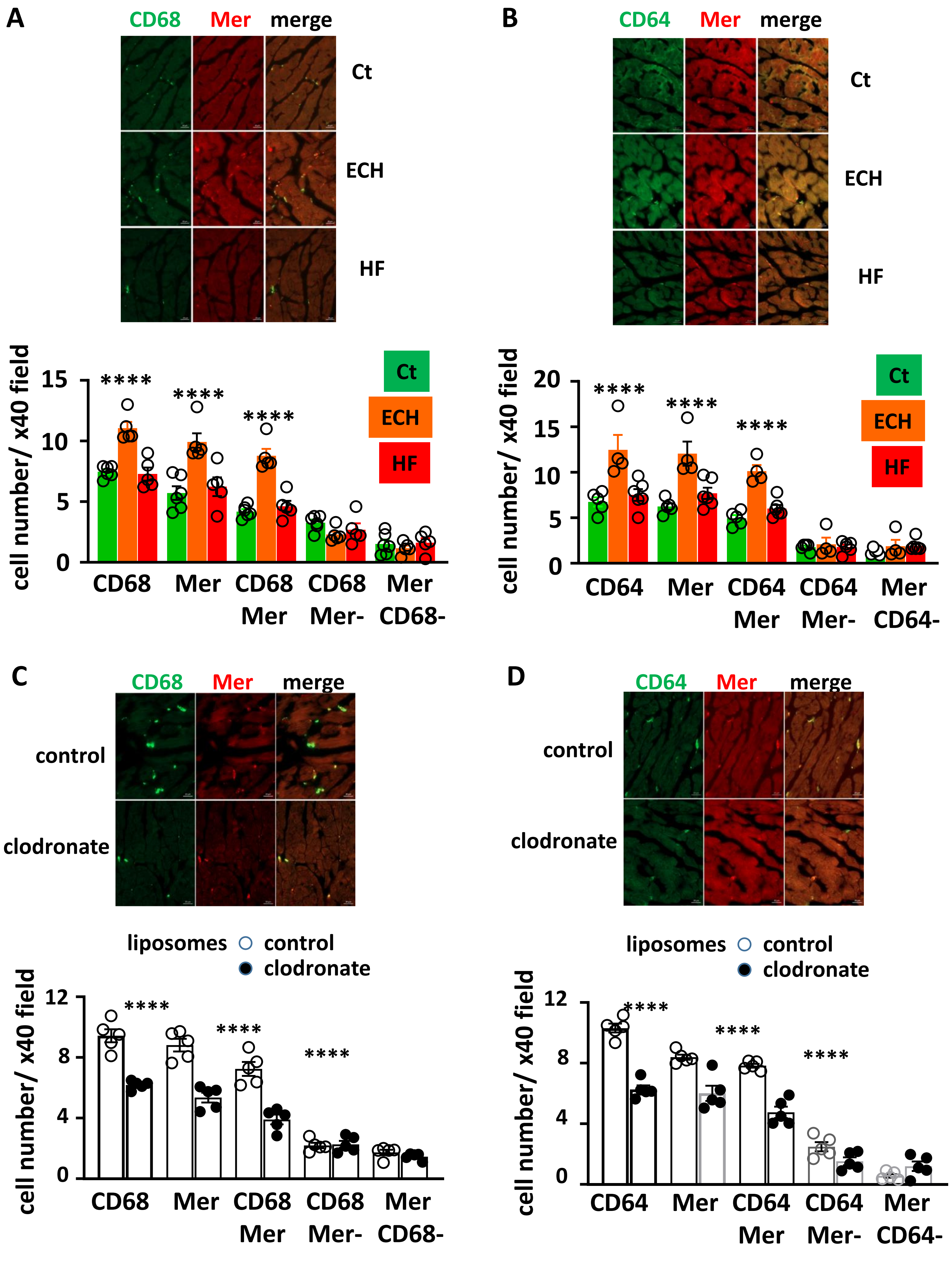

Figure S6: **(A and B) Isoproterenol infusion elicits a selective early transient increase in cardiac CD68$^+$/Mer$^+$, CD64$^+$/Mer$^+$ Mφ. (C and D) Efficient depletion of cardiac CD68$^+$/Mer$^+$ and CD64$^+$/Mer$^+$ Mφ upon clodronate treatment.** Typical images of cardiac sections stained for cardiac CD68$^+$ or CD64$^+$ and Mer$^+$ and quantification of the different cell number; n=4-6 mice/group, 32 images/mice, Kruskal-Wallis followed by Dunn's post-hoc test; * $p<0.05$. scale bar 20µm.

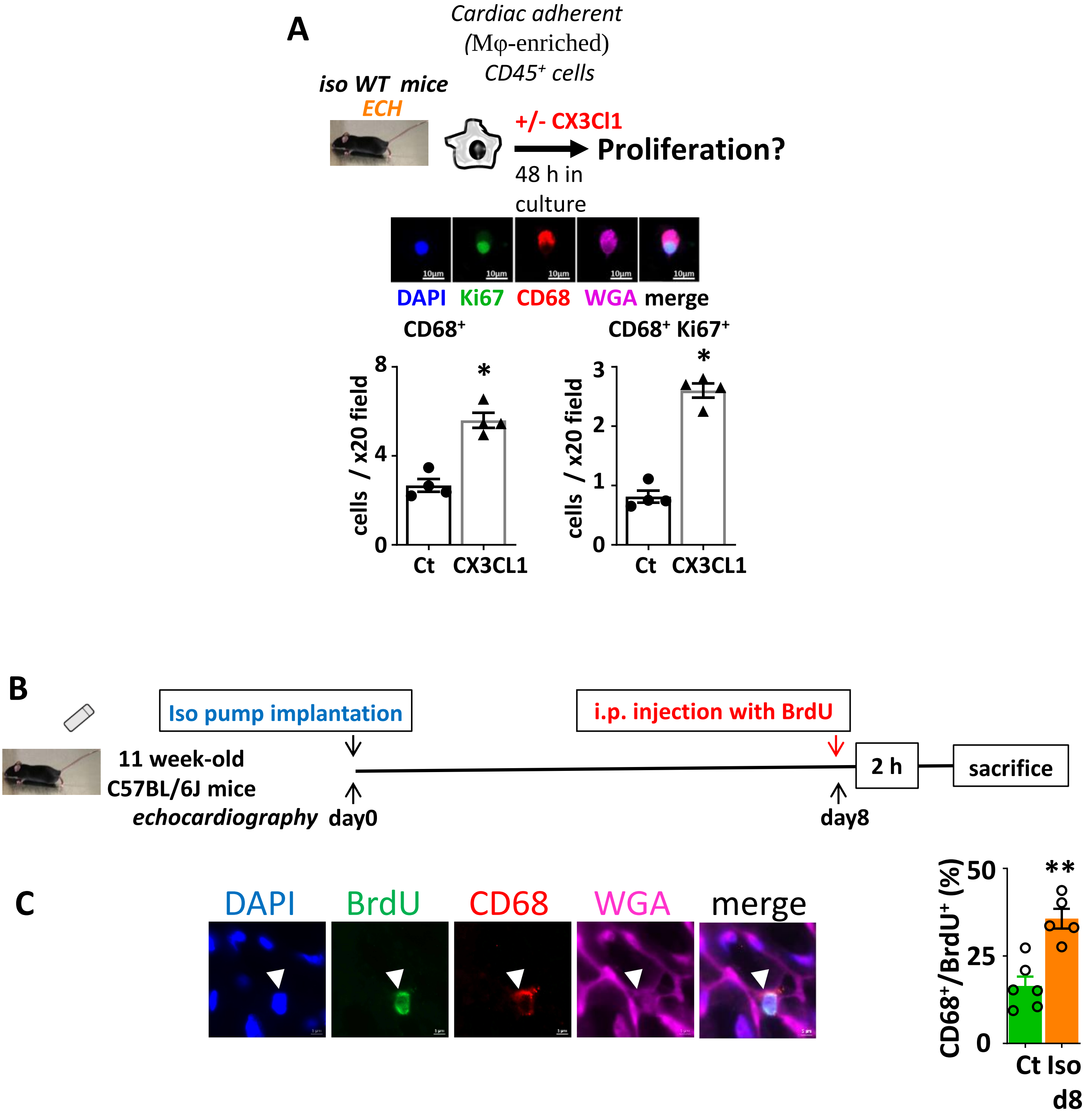

Figure S7: **Mφ proliferation is elicited by CX3CL1 *in-vitro* and detected in early Iso-infused hearts *in-vivo.*** **(A)** CX3CL1 enhances the number and proliferative activity of Mφ-enriched CD45$^+$ cells isolated from iso-infused ECH hearts *in-vitro*. Typical images of immune cells stained for DAPI, CD68, Ki67 and WGA and quantification of CD68$^+$ and CD68$^+$ Ki67$^+$ cell number. Mean±SEM from quadruplicate. Cells were isolated from 5 iso-infused mice, sacrificed at day 8, Mann-Whitney U test; *p<0.05. **(B)** Mφ proliferation in iso-infused hearts, attested by *in-vivo* BrdU incorporation. Schematic representation of the BrDU injection protocol. **(C)** Typical images of cardiac sections stained for DAPI, CD68, BrdU and WGA and quantification of CD68$^+$ BrdU$^+$ cell number. Mean±SEM from n=6 (Ct) and n=5 (iso-infused) mice, sacrificed at day 8, Mann-Whitney U test; **p<0.01

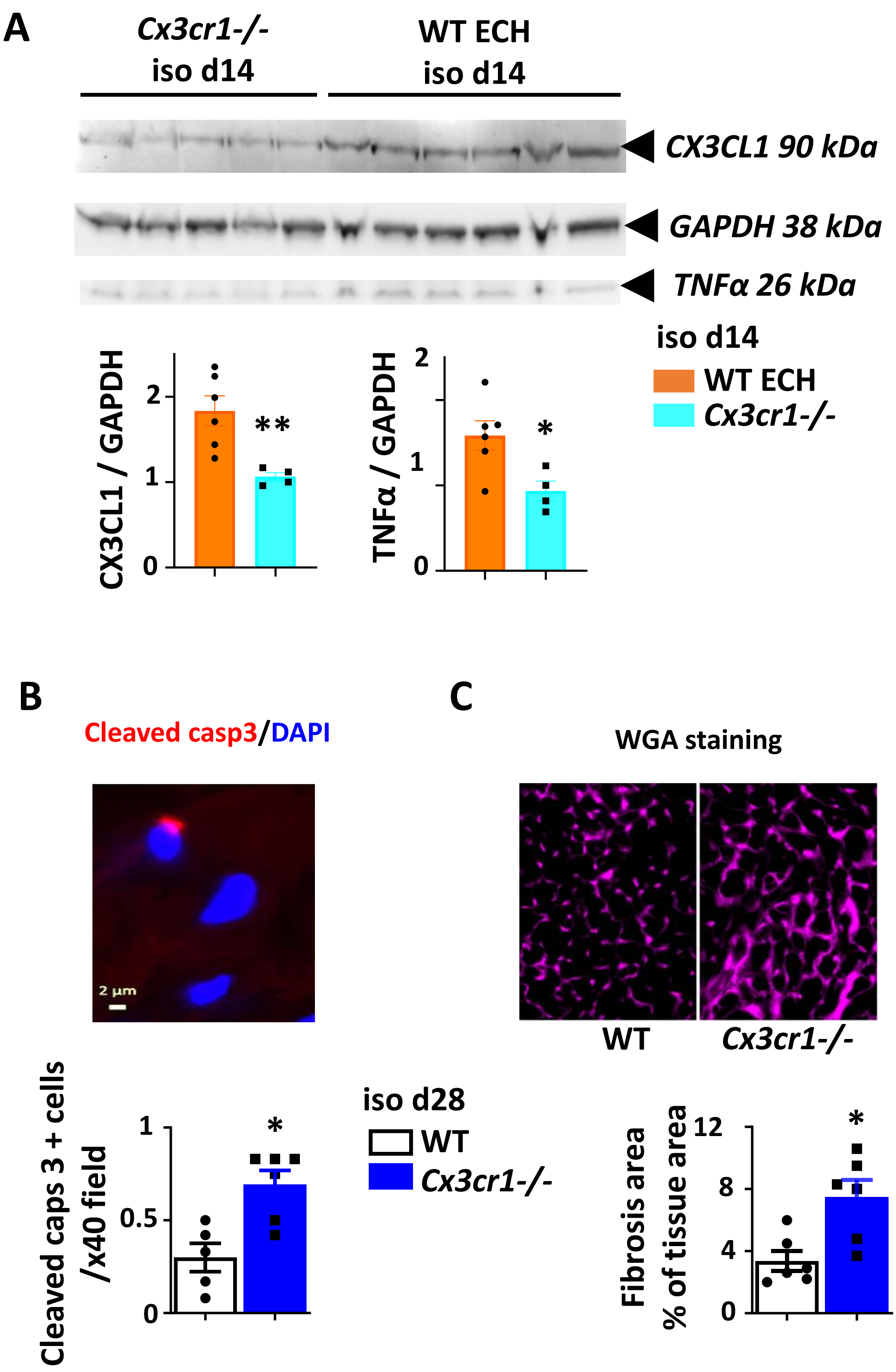

Figure S8: **Iso-infused *Cx3cr1* $^{-/-}$ hearts display lower levels of CX3CL1 and TNFα at day 14 and higher apoptosis and fibrosis at day 28, as compared to WT hearts. (A)** CX3CL1 and TNFα levels in WT ECH or Cx3cr1$^{-/-}$ hearts infused for 14 days with iso. Full unedited gels are provided at the end of the supplementary material as well as detailed procedure (Figure S11). Mean±SEM from 5-6 mice/group, Mann Withney test; * p<0.05, ** p<0.01. **(B)** Apoptosis estimated in cardiac sections stained with cleaved caspase 3 Abs (typical image). Mean±SEM from 6 mice/group, sacrificed at day28, Mann-Whitney U test; **p*<0.05. **(C)** Fibrosis area estimated in cardiac sections stained with WGA (typical image). Mean±SEM from 6 mice/group, sacrificed at day28, Mann-Whitney U test; **p*<0.05.

Intramyocardial injection under echography guidance

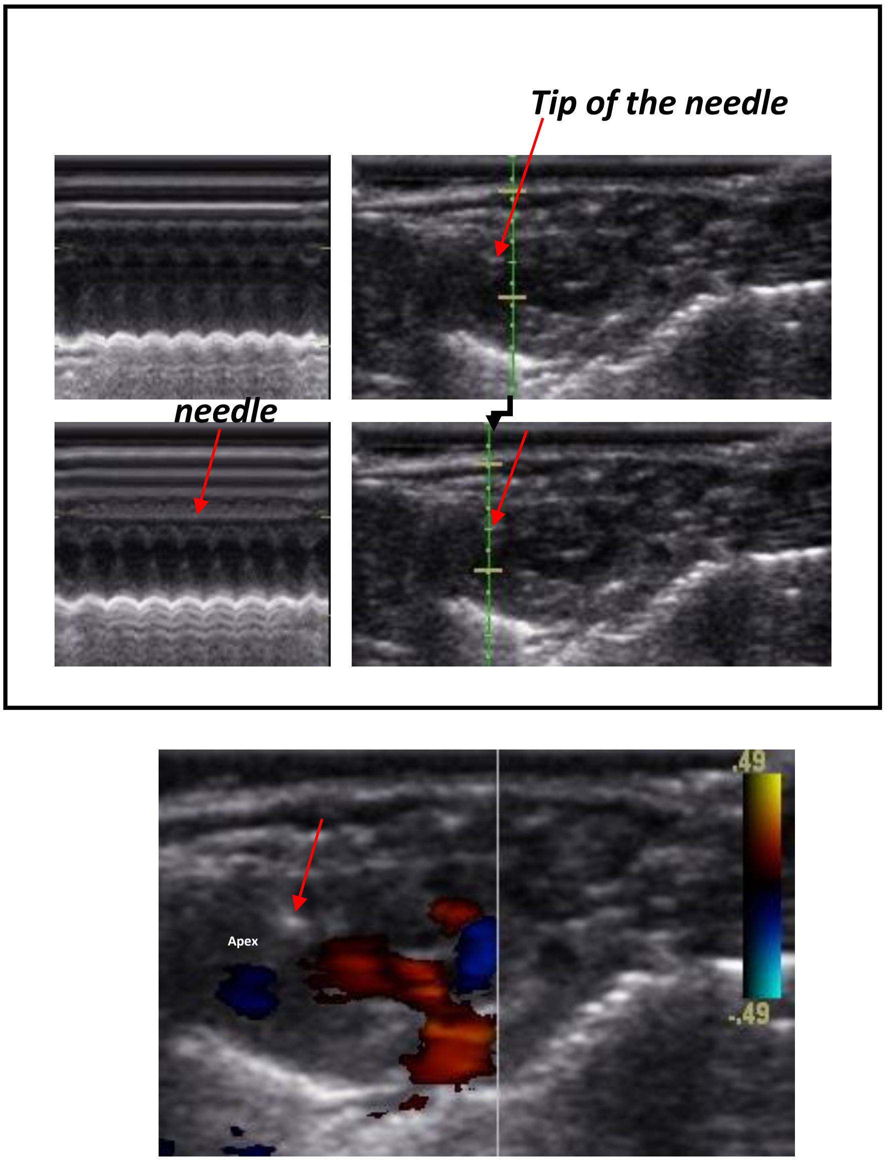

Figure S9: **Visualization of the tip of the needle during intramyocardial injection under echocardiography guidance.**

Supplementary information

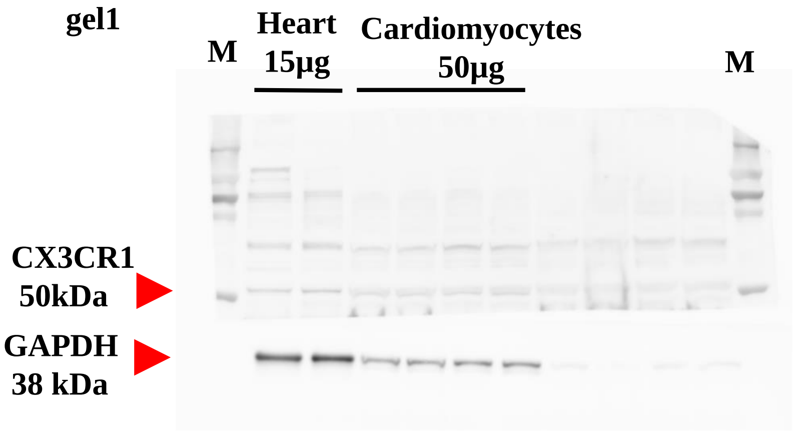

Full unedited gels with membrane edge visible (expo 10 sec 4) to Figure 4A: Blots of Figure 4A were obtained from lane 2 and 3 gel 1 and results reproduced from other samples in gel 2. Nitrocellulose membranes were cut. Upper part of membrane was incubated with anti-CX3CR1 Ab, and lower part with anti-GAPDH Ab, followed by HRP Ab. Detection of ECL signals was performed after reassembling of membrane parts and was recorded using a Camera LAS 4000.

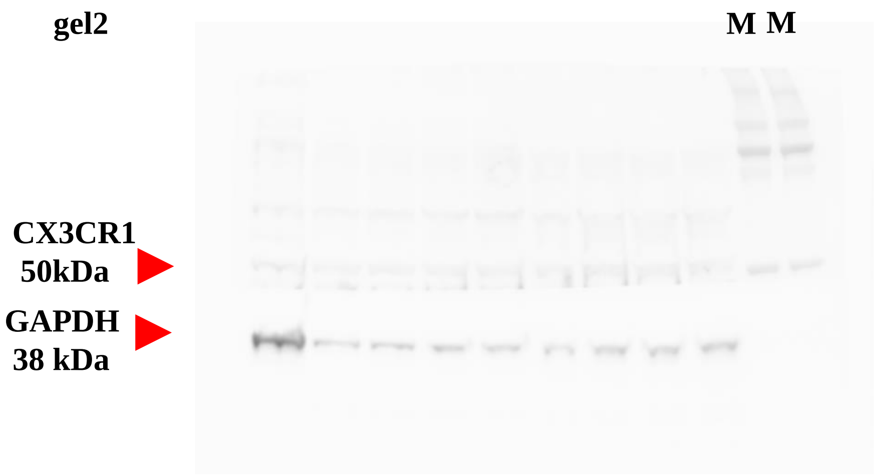

Figure S10

Supplementary information

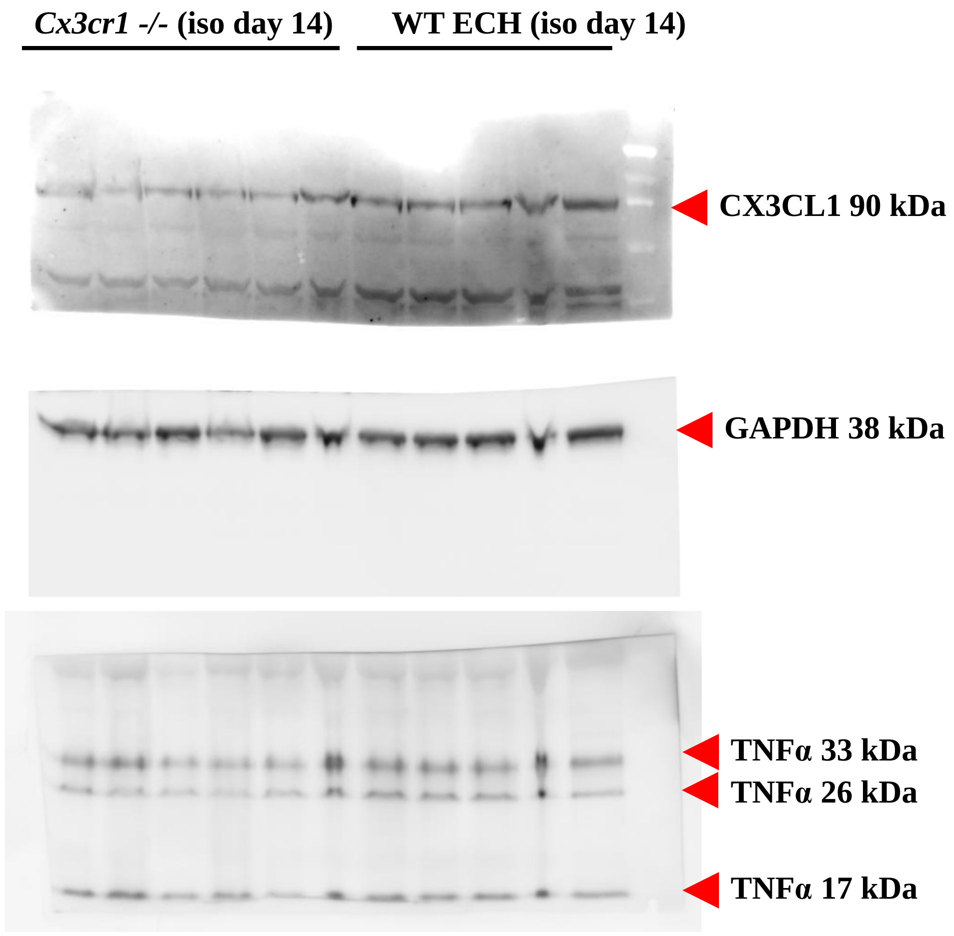

Full unedited gels to Figure S8A: Nitrocellulose membrane was cut. Upper part of membrane was incubated with anti-CX3CL1 Ab, and lower part with anti-TNFα Ab before stripping and hybridization with anti-GAPDH Ab.

Figure S11

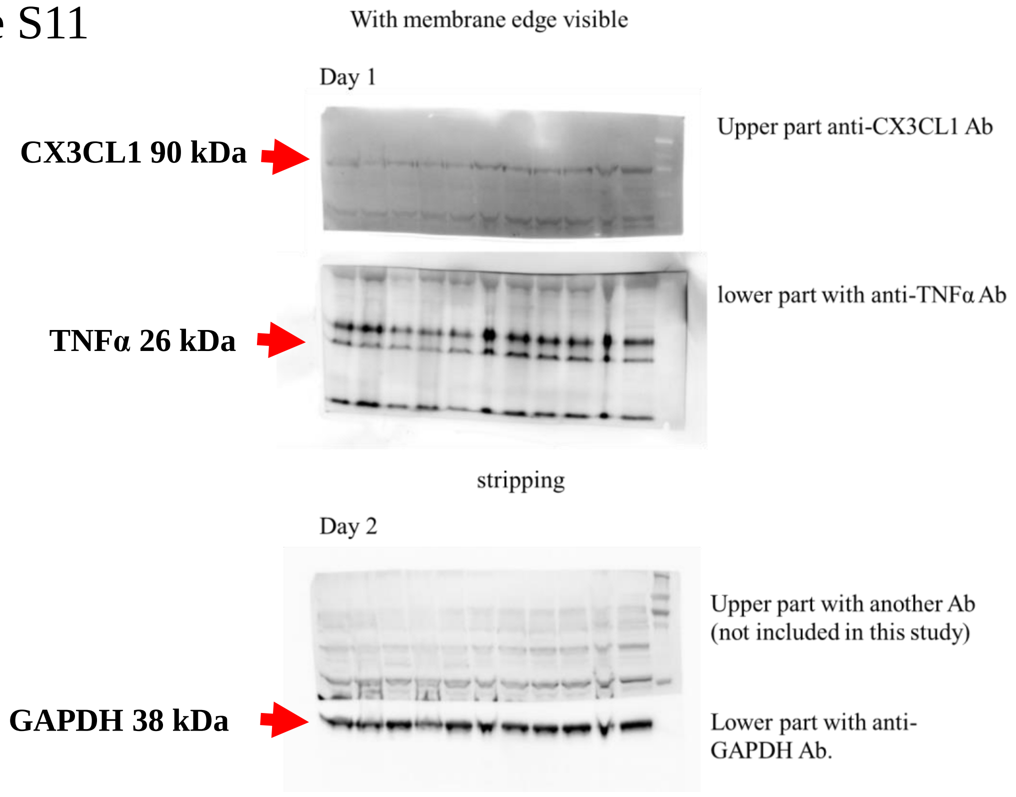

Supplementary information

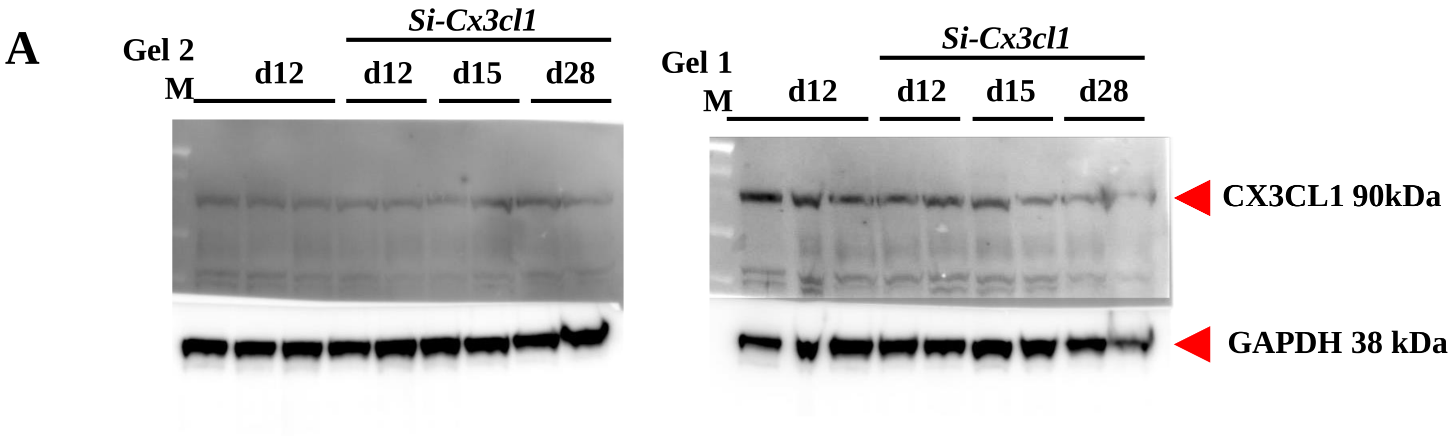

<u>Full unedited gels to Figure 6C:</u> Blots of Figure 4C were obtained from gel 1 (A, right panel) and experiment reproduced from other samples in gel 2 (A, left panel). Nitrocellulose membranes were cut. Upper part of membrane was incubated with anti-CX3CL1 Ab, and lower part with anti-GAPDH Ab, followed by HRP Ab. Detection of ECL signals was performed after reassembling of membrane parts and was recorded using a Camera LAS 4000.

With membrane edge visible

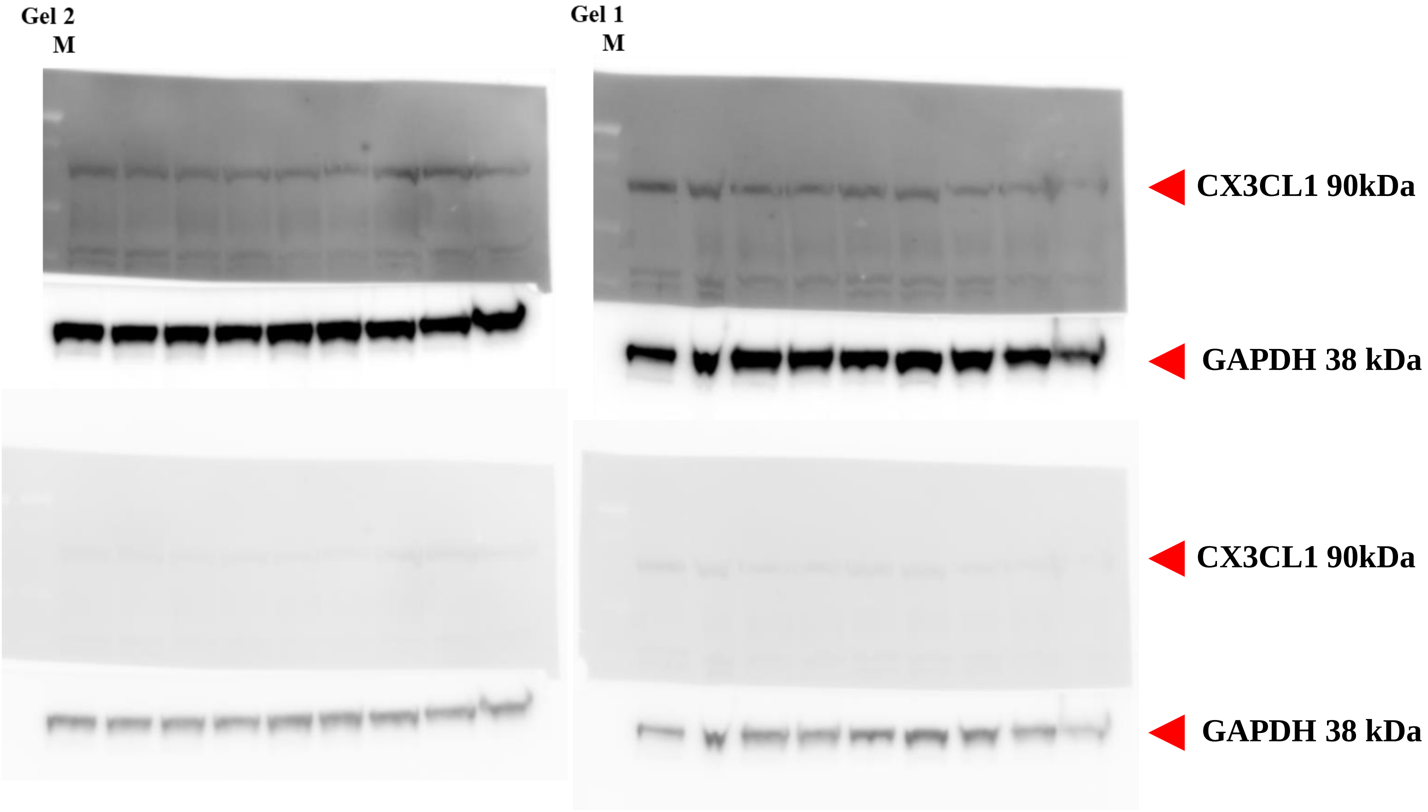

Figure S12

Supplementary information

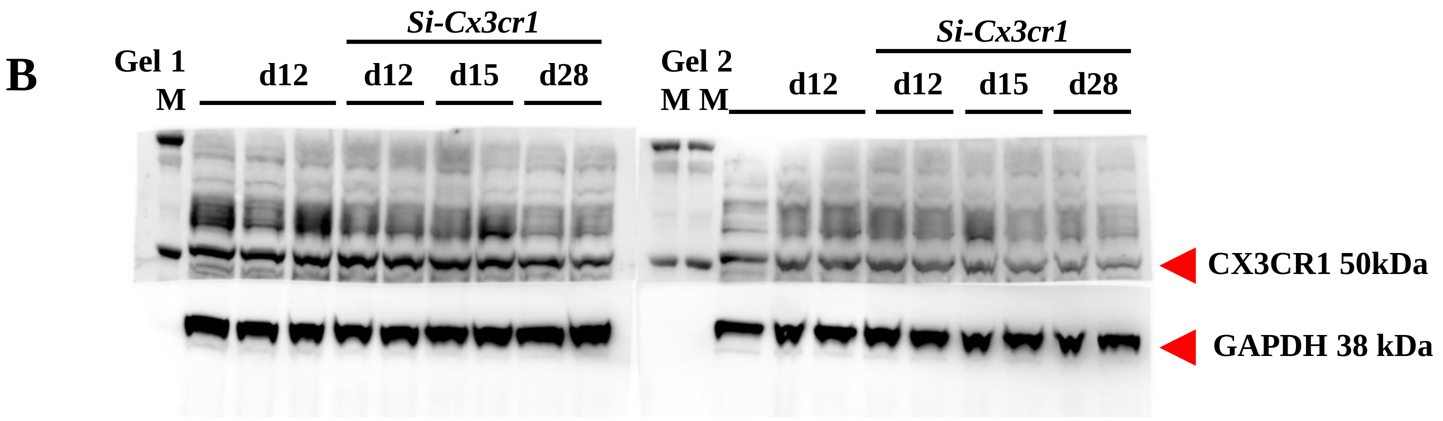

Full unedited gels to Figure 6F: Blots of Figure 6F were obtained from gel 1 (B, left panel) and experiment reproduced from other samples in gel 2 (B, right panel). Nitrocellulose membranes were cut. Upper part of membrane was incubated with anti-CX3CR1 Ab, and lower part with anti-GAPDH Ab, followed by HRP Ab. Detection of ECL signals was performed after reassembling of membrane parts and was recorded using a Camera LAS 4000.

With membrane edge visible

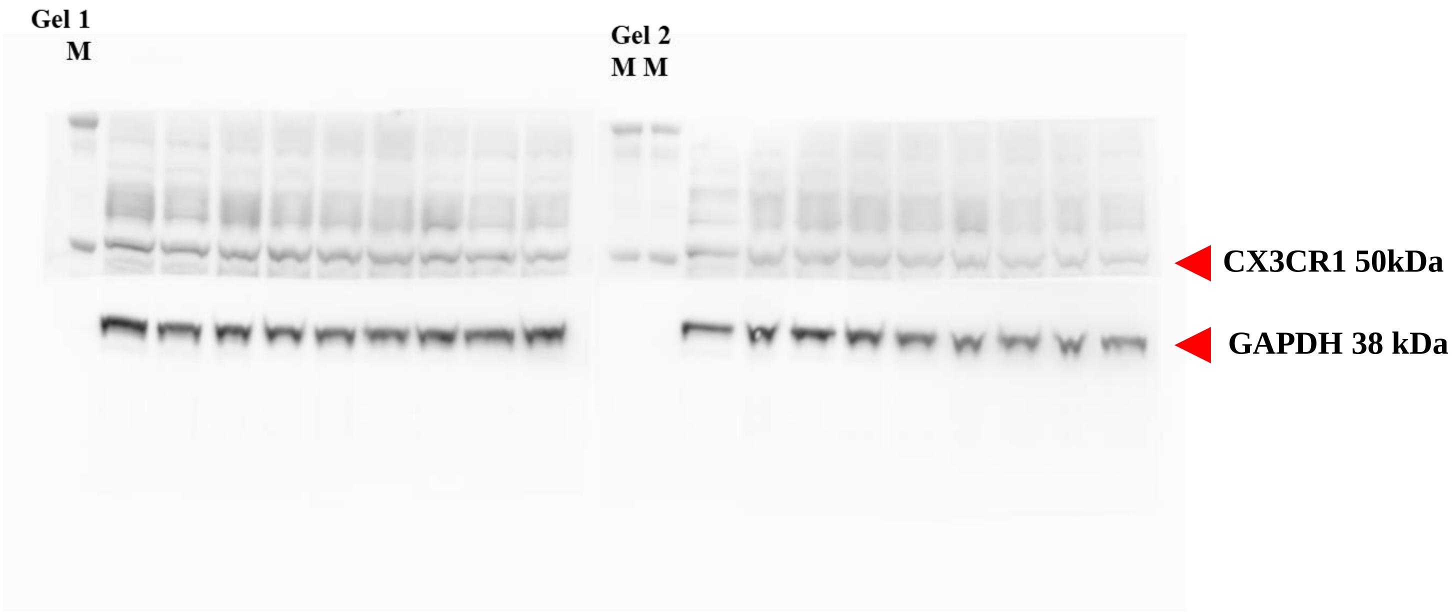

Figure S13

Parameter	Ctl (n=8)<br>ctl	Iso at d14 (n=11)<br>ECH	Iso at d28 (n=7)<br>HF
HR (bpm)	610±14	646±9	648±22
IVSd (mm)	0.64±0.3	0.9±0.03 *†	0.65±0.02
LVd (mm)	3.53±0.08	3.67±0.12 †	4.4±0.1
PWd (mm)	0.64±0.03	0.79±0.03 †	0.54±0.02
IVSs (mm)	1.08±0.044	1.36±0.036 * †	0.94±0.02
LVs (mm)	1.92±0.04	2±0.11 †	3.28±0.82
PWs (mm)	1.11±0.02	1.25±0.01 * †	0.84±0.04
h/r	0.37±0.009	0.48±0.02 * †	0.27±0.01
EF (%)	82.82±0.56	82.74±1.23 †	57.17±0.99
FS (%)	45.56±0.61	45.75±1.25 †	25.66±0.6

Table S1: **Echocardiography parameters at day 14 and day 28 in mice implanted or not with Iso pump at day 0.** Kruskal-Wallis followed by Dunn post-hoc tests. * $p<0.05$ ECH vs ctl; † $p<0.05$ ECH vs HF.

HR, heart rate; IVSd, end-diastolic interventricular septum thickness; LVd, end-diastolic left ventricular diameter; PWd, end-diastolic posterior wall thickness; IVSs, end-systolic interventricular septum thickness; LVs, end-systolic left ventricular diameter; PWs, end-systolic posterior wall thickness; h/r, diastolic wall thickness to radius ratio; EF, ejection fraction; FS, fractional shortening.

Iso mice (iso pump implantation at d0)															
Time of echocardiography	**d0**			**d9**			**d14**			**d21**			**d25**		
1st injection at d7	**control (n=9)**	**clodro (n=4)**	***p***	**control (n=9)**	**clodro (n=4)**	***p***	**control (n=9)**	**clodro (n=4)**	***p***	**control (n=9)**	**clodro (n=4)**	***p***	**control (n=9)**	**clodro (n=4)**	***p***
HR (bpm)	618± 4	616± 11	*ns*	650± 14	645± 16	*ns*	652± 13	628± 13	*ns*	626± 13	598± 5	*ns*	625± 10	596± 15	*ns*
IVSd (mm)	0.6± 0.01	0.6± 0.02	*ns*	1± 0.03	0.9± 0.03	*ns*	0.9± 0.04	0.8± 0.04	*ns*	0.8± 0.04	0.8± 0.07	*ns*	0.9± 0.04	0.8± 0.02	*ns*
LVd (mm)	3.5± 0.04	3.5± 0.05	*ns*	3.7± 0.22	3.8± 0.11	*ns*	3.8± 0.26	4.2± 0.08	*ns*	4.1± 0.22	3.6± 0.18	*ns*	4 ± 0.25	3.5± 0.35	*ns*
PWd (mm)	0.7± 0.03	0.7± 0.04	*ns*	**1± 0.03**	**0.8± 0.02**	***<0.05***	**0.8± 0.03**	**0.6± 0.07**	***<0.01***	0.7± 0.04	0.6± 0.02	*ns*	0.8± 0.03	0.7± 0.02	*ns*
IVSs (mm)	1.1± 0.02	1.1± 0.02	*ns*	1.4± 0.06	1.3± 0.04	*ns*	1.3± 0.06	1.1± 0.05	*ns*	1.2± 0.06	1.0± 0.04	*ns*	1.2± 0.06	1.2± 0.04	*ns*
LVs (mm)	1.9± 0.04	1.9± 0.03	*ns*	2.2± 0.3	2.1± 0.1	*ns*	2.4± 0.32	2.9± 0.18	*ns*	2.7± 0.3	2.2± 0.16	*ns*	2.6± 0.34	2.3± 0.13	*ns*
PWs (mm)	1.1± 0.04	1.1± 0.04	*ns*	1.3± 0.07	1.2± 0.07	*ns*	**1.3± 0.06**	**0.9± 0.09**	***<0.05***	1.1± 0.09	1.0± 0.06	*ns*	1.1± 0.07	1.1± 0.06	*ns*
h/r	0.378± 0.009	0.380± 0.009	*ns*	0.552± 0.034	0.44± 0.017	*ns*	0.483± 0.042	0.34± 0.024	*ns*	0.388± 0.03	0.39± 0.045	*ns*	0.42± 0.036	0.46± 0.025	*ns*
EF (%)	83.2± 0.4	84.3± 0.26	*ns*	78.5± 5	81.02± 1.1	*ns*	73.6± 5.8	65.02± 3.93	*ns*	69.39± 5.8	74.2± 1.8	*ns*	70.9± 6.04	73.08± 3.3	*ns*
FS (%)	45.96± 0.41	47.18± 0.29	*ns*	43.64± 3.89	43.78± 1.16	*ns*	39.42± 4.15	31.03± 2.67	*ns*	35.66± 3.81	37.51± 1.48	*ns*	37.04± 3.97	36.85± 2.59	*ns*

Table S2: **Echocardiography parameters at day 0, 9, 14, 21 and day 25 in mice implanted with Iso pump at day 0 and with the first injection of control/clodronate liposomes at day 7**. n=4-9 mice/group, two-way ANOVA followed by Sidak's post-hoc tests. *HR, heart rate; IVSd, end-diastolic interventricular septum thickness; LVd, end-diastolic left ventricular diameter; PWd, end-diastolic posterior wall thickness; IVSs, end-systolic interventricular septum thickness; LVs, end-systolic left ventricular diameter; PWs, end-systolic posterior wall thickness; h/r, diastolic wall thickness to radius ratio; EF, ejection fraction; FS, fractional shortening.*

Iso mice (iso pump implantation at d0)									
Time of echocardiography	**d0**			**d14**			**d28**		
	**WT (n=33)**	***Cx3cr1-/-* (n=38)**	***p***	**WT (n=17)**	***Cx3cr1-/-* (n=27)**	***p***	**WT (n=14)**	***Cx3cr1-/-* (n=24)**	***p***
HR (bpm)	625± 4	600± 3	*ns*	637± 7	622± 8	*ns*	636± 9	620± 12	*ns*
IVSd (mm)	0.62± 0.01	0.65± 0.01	*ns*	0.96± 0.02	0.81± 0.02	*<0.01*	0.87± 0.03	0.75± 0.02	*<0.05*
LVd (mm)	3.53± 0.03	3.66± 0.05	*ns*	3.52± 0.11	4.21± 0.09	*<0.0001*	3.74± 0.10	4.3± 0.09	*<0.01*
PWd (mm)	0.63± 0.02	0.59± 0.02	*ns*	0.86± 0.04	0.69± 0.02	*<0.001*	0.78± 0.03	0.63± 0.03	*<0.001*
IVSs (mm)	1.09± 0.02	1.14± 0.01	*ns*	1.39± 0.03	1.19± 0.03	*<0.0001*	1.27± 0.06	1.19± 0.04	*ns*
LVs (mm)	1.92± 0.02	1.99± 0.03	*ns*	1.91± 0.09	2.67± 0.1	*<0.0001*	2.32± 0.15	2.89± 0.12	*<0.01*
PWs (mm)	1.1± 0.02	1.11± 0.02	*ns*	1.28± 0.04	1.13± 0.03	*<0.01*	1.11± 0.05	0.99± 0.04	*<0.05*
h/r	0.358± 0.005	0.343± 0.007	*ns*	0.528± 0.024	0.362± 0.014	*<0.0001*	0.451± 0.026	0.328± 0.014	*<0.0001*
EF (%)	83.2± 0.2	82.8± 0.3	*ns*	83± 1	73.1± 1.5	*<0.0001*	74.2± 2.9	68± 2.1	*<0.05*
FS (%)	46± 0.2	45.5± 0.3	*ns*	46.0± 1	37.2± 1.1	*<0.0001*	38.8± 2.4	33.4± 1.4	*<0.05*

Table S3: **Kinetics of echocardiography parameters in WT or *Cx3cr1-/-* mice implanted with Iso pump at day 0.** n=14-38 mice, Two-way ANOVA followed by Sidak's post-hoc tests.

HR, heart rate; IVSd, end-diastolic interventricular septum thickness; LVd, end-diastolic left ventricular diameter; PWd, end-diastolic posterior wall thickness; IVSs, end-systolic interventricular septum thickness; LVs, end-systolic left ventricular diameter; PWs, end-systolic posterior wall thickness; h/r, diastolic wall thickness to radius ratio; EF, ejection fraction; FS, fractional shortening.

Iso mice (iso pump implantation at d0)																		
**Time of echocardiography**	**d0**			**d7**			**d12**			**d15**			**d22**			**d28**		
**Injection at d7**	**siScr (n=13)**	**si*Cx3cl1* (n=16)**	p	**siScr (n=12)**	**si*Cx3cl1* (n=15)**	p	**siScr (n=12)**	**si*Cx3cl1* (n=15)**	p	**siScr (n=10)**	**si*Cx3cl1* (n=11)**	p	**siScr (n=10)**	**si*Cx3cl1* (n=8)**	p	**siScr (n=5)**	**si*Cx3cl1* (n=8)**	p
**HR (bpm)**	635± 6	635± 5	ns	668± 9	179± 12	ns	652±8	660± 10	ns	663± 10	662± 13	ns	648± 8	628± 6	ns	656± 18	669± 7	ns
**IVSd (mm)**	0.67± 0.01	0.67± 0.01	ns	1.06± 0.02	1± 0.02	ns	0.93± 0.02	0.99± 0.03	ns	0.95± 0.02	0.88± 0.03	<0.05	0.87± 0.03	0.84± 0.03	ns	0.92± 0.02	1± 0.03	ns
**LVd (mm)**	3.46± 0.04	3.44± 0.05	ns	3.33± 0.8	3.42± 0.1	ns	3.51± 0.09	3.61± 0.08	ns	3.5± 0.06	3.75± 0.1	ns	3.73± 0.14	4.11± 0.14	ns	3.5± 0.21	3.52± 0.11	ns
**PWd (mm)**	0.65± 0.01	0.62± 0.02	ns	0.9± 0.02	0.83± 0.02	<0.05	0.78± 0.03	0.8± 0.03	ns	0.8± 0.03	0.67± 0.03	<0.01	0.78± 0.02	0.76± 0.03	ns	0.82± 0.04	0.81± 0.04	ns
**IVSs (mm)**	1.15± 0.02	1.12± 0.03	ns	1.54± 0.03	1.68± 0.1	ns	1.37± 0.04	1.49± 0.03	<0.05	1.39± 0.03	1.26± 0.05	ns	1.32± 0.04	1.26± 0.07	ns	1.3± 0.04	1.42± 0.03	ns
**LVs (mm)**	1.85± 0.03	1.81± 0.03	ns	1.68± 0.09	1.77± 0.08	ns	1.92± 0.1	1.96± 0.1	ns	1.98± 0.06	2.31± 0.12	<0.05	2.08± 0.17	2.68± 0.19	<0.05	1.84± 0.18	2.01± 0.16	ns
**PWs (mm)**	1.15± 0.01	1.1± 0.02	<0.05	1.37± 0.04	1.29± 0.03	ns	1.28± 0.06	1.3± 0.03	ns	1.28± 0.04	1.11± 0.04	<0.001	1.29± 0.04	1.15± 0.04	<0.05	1.3± 0.04	1.29± 0.06	ns
**h/r**	0.38± 0.007	0.37± 0.007	ns	0.59± 0.018	0.55± 0.018	ns	0.49± 0.02	0.5± 0.019	ns	0.50± 0.017	0.42± 0.015	<0.01	0.45± 0.026	0.4± 0.027	ns	0.50± 0.027	0.52± 0.022	ns
**EF (%)**	84± 0.2	84.1± 0.3	ns	86.1± 1.6	85± 1.1	ns	82.2± 1.6	82.1± 1.9	ns	80.9± 1	74.3± 2.4	<0.05	81.3± 2.6	70.3± 3.4	<0.05	84.4± 1.9	79.5± 3	ns
**FS (%)**	46.8± 0.2	47.1± 0.3	Ns	50± 1.7	48.4± 1.3	ns	45.5± 1.6	45.7± 1.9	ns	43.7± 1	40.9± 2.8	ns	44.9± 2.3	36.1± 2.9	<0.05	47.7± 2.1	42.8± 3.1	ns

Table S4: **Echocardiography parameters at day 0, 7, 12, 15, 21 and day 28 in mice implanted with Iso pump at day 0 and with the injection of siScramble or si*Cx3cl1* RNA at day 7.** n=5-16 mice/group, two-way ANOVA followed by Sidak's post-hoc tests.

HR, heart rate; IVSd, end-diastolic interventricular septum thickness; LVd, end-diastolic left ventricular diameter; PWd, end-diastolic posterior wall thickness; IVSs, end-systolic interventricular septum thickness; LVs, end-systolic left ventricular diameter; PWs, end-systolic posterior wall thickness; h/r, diastolic wall thickness to radius ratio; EF, ejection fraction; FS, fractional shortening.

Iso mice (iso pump implantation at d0)												
**Time of echocardiography**	**d0**			**d7**			**d12**			**d15**		
**Injection at d7**	**siScr (n=10)**	**si*Cx3cr1* (n=14)**	p	**siScr (n=10)**	**si*Cx3cr1* (n=14)**	p	**siScr (n=10)**	**si*Cx3cr1* (n=14)**	p	**siScr (n=10)**	**si*Cx3cr1* (n=14)**	p
**HR (bpm)**	622± 5	635± 5	ns	684±10	672± 9	ns	668±9	667± 8	ns	674± 11	671± 6	ns
**IVSd (mm)**	0.63± 0.01	0.66± 0.01	ns	1.04± 0.02	1.06± 0.02	ns	1.01± 0.03	0.96± 0.02	ns	0.93± 0.01	0.95± 0.03	ns
**LVd (mm)**	3.56± 0.05	3.41± 0.06	ns	3.28± 0.08	3.40± 0.1	ns	3.77± 0.15	3.71± 0.17	ns	3.56± 0.1	3.69± 0.17	ns
**PWd (mm)**	0.63± 0.03	0.61± 0.03	ns	0.89± 0.03	0.88± 0.03	ns	0.87± 0.06	0.74± 0.03	ns	0.78± 0.04	0.76± 0.04	ns
**IVSs (mm)**	1.14± 0.03	1.12± 0.03	ns	1.52± 0.04	1.56± 0.04	ns	1.47± 0.02	1.36± 0.04	ns	1.4± 0.03	1.39± 0.04	ns
**LVs (mm)**	1.93± 0.03	1.81± 0.04	ns	1.65± 0.11	1.76± 0.11	ns	1.67± 0.05	2.25± 0.2	<0.05	1.97± 0.11	2.20± 0.21	ns
**PWs (mm)**	1.17± 0.01	1.11± 0.02	ns	1.41± 0.05	1.34± 0.03	ns	1.42± 0.05	1.21± 0.06	<0.05	1.29± 0.04	1.19± 0.07	ns
**h/r**	0.37± 0.01	0.37± 0.01	ns	0.59± 0.02	0.58± 0.02	ns	0.56± 0.03	0.47± 0.03	<0.05	0.49± 0.02	0.48± 0.03	ns
**EF (%)**	83.6± 0.2	83.9± 0.3	ns	86.2± 2	84.3± 2.15	ns	86.9± 0.5	76.01± 3.3	<0.01	82.3± 1.6	76.9± 3.4	ns
**FS (%)**	46.4± 0.3	46.8± 0.3	ns	50.2± 2.1	48.4± 2.1	ns	50.4± 0.7	40.6± 2.7	<0.01	45.4± 1.6	41.6± 2.9	ns

Table S5**: Echocardiography parameters at day 0, 7, 12 and day 15 in mice implanted with Iso pump at day 0 and with the injection of siScramble or si*Cx3cr1* at day 7.** n=10-14 mice/group, two-way ANOVA followed by Sidak's post-hoc tests.

HR, heart rate; IVSd, end-diastolic interventricular septum thickness; LVd, end-diastolic left ventricular diameter; PWd, end-diastolic posterior wall thickness; IVSs, end-systolic interventricular septum thickness; LVs, end-systolic left ventricular diameter; PWs, end-systolic posterior wall thickness; h/r, diastolic wall thickness to radius ratio; EF, ejection fraction; FS, fractional shortening.
